# Comparative genomics of *Chlamydomonas*

**DOI:** 10.1093/plcell/koab026

**Published:** 2021-02-02

**Authors:** Rory J Craig, Ahmed R Hasan, Rob W Ness, Peter D Keightley

**Affiliations:** School of Biological Sciences, Institute of Evolutionary Biology, University of Edinburgh, EH9 3FL Edinburgh, UK; Department of Biology, University of Toronto Mississauga, Mississauga, Onatrio, Canada L5L 1C6; Department of Biology, University of Toronto Mississauga, Mississauga, Onatrio, Canada L5L 1C6; Department of Biology, University of Toronto Mississauga, Mississauga, Onatrio, Canada L5L 1C6; School of Biological Sciences, Institute of Evolutionary Biology, University of Edinburgh, EH9 3FL Edinburgh, UK

## Abstract

Despite its role as a reference organism in the plant sciences, the green alga *Chlamydomonas reinhardtii* entirely lacks genomic resources from closely related species. We present highly contiguous and well-annotated genome assemblies for three unicellular *C. reinhardtii* relatives: *Chlamydomonas incerta*, *Chlamydomonas schloesseri*, and the more distantly related *Edaphochlamys debaryana*. The three *Chlamydomonas* genomes are highly syntenous with similar gene contents, although the 129.2 Mb *C. incerta* and 130.2 Mb *C. schloesseri* assemblies are more repeat-rich than the 111.1 Mb *C. reinhardtii* genome. We identify the major centromeric repeat in *C. reinhardtii* as a LINE transposable element homologous to *Zepp* (the centromeric repeat in *Coccomyxa subellipsoidea*) and infer that centromere locations and structure are likely conserved in *C. incerta* and *C. schloesseri*. We report extensive rearrangements, but limited gene turnover, between the *minus* mating type loci of these *Chlamydomonas* species. We produce an eight-species core-*Reinhardtinia* whole-genome alignment, which we use to identify several hundred false positive and missing genes in the *C. reinhardtii* annotation and >260,000 evolutionarily conserved elements in the *C. reinhardtii* genome. In summary, these resources will enable comparative genomics analyses for *C. reinhardtii*, significantly extending the analytical toolkit for this emerging model system.

## Teaser

High-quality genome assemblies and annotations for three of the closest relatives of *Chlamydomonas reinhardtii* enable comparative genomics analyses.

##  

**Figure:**
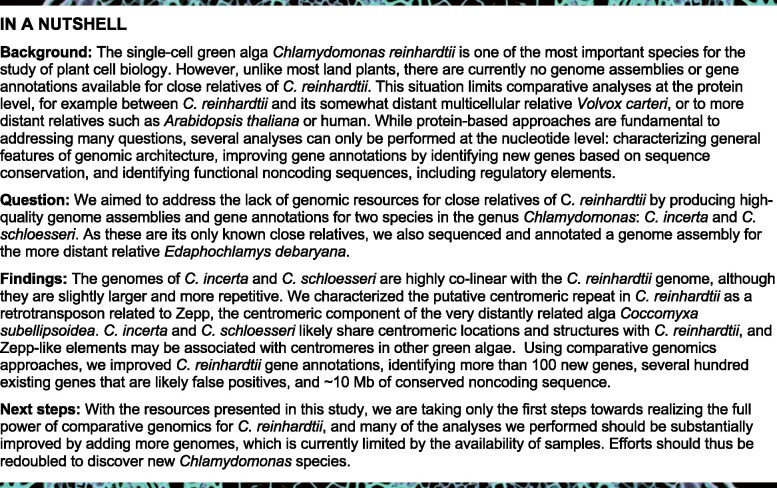


## Introduction

With the rapid increase in genome sequencing over the past two decades, comparative genomics analyses have become a fundamental tool in biological research. As the first sets of genomes for closely related eukaryotic species became available, pioneering comparative studies led to refined estimates of gene content and orthology, provided deeper understanding of the evolution of genome architecture and the extent of genomic synteny between species, and enabled the proportions of genomes evolving under evolutionary constraint to be estimated for the first time (Mouse Genome Sequencing Consortium, 2002; Cliften et al., 2003; Stein et al., 2003; Richards et al., 2005). As additional genomes were sequenced, it became possible to produce whole-genome alignments (WGAs) across multiple species and to identify conserved elements (CEs) in noncoding regions for several of the most well-studied lineages (Siepel et al., 2005; Stark et al., 2007; Gerstein et al., 2010; Lindblad-Toh et al., 2011). Many of these conserved noncoding sequences overlap regulatory elements and the identification of CEs has proven to be among the most accurate approaches for discovering functional genomic sequences (Alföldi and Lindblad-Toh, 2013). WGAs are also powerful resources for directly improving gene annotations, with applications including the identification of novel genes, splice forms, and exons (Lin et al., 2007; Mudge et al., 2019), distinguishing between protein-coding and long noncoding RNA (lncRNA) loci (Pauli et al. 2012), and the identification of non-standard protein-coding features such as translational frameshifts and stop codon readthrough (Lin et al., 2007; Jungreis et al., 2011).

The ability to perform comparative analyses is contingent on the availability of genome assemblies for species that span a range of appropriate evolutionary distances. While this state has been achieved for the majority of model organisms, there remain several species of high biological significance that entirely lack genomic resources for any closely related species. Hiller et al. (2013) described such cases as “phylogenetically isolated genomes,” specifically referring to species for which the most closely related sequenced genomes belong to species divergent by one or more substitutions, on average, per neutrally evolving site. At this scale of divergence, an increasingly negligible proportion of the genome can be aligned at the nucleotide level (Margulies et al., 2006), thereby limiting comparative analyses at the protein-level and impeding the development of such species as model systems in numerous research areas.

The unicellular green alga *Chlamydomonas reinhardtii* is a long-standing reference organism for several fields, including cell biology, plant physiology and algal biotechnology (Salomé and Merchant, 2019). Because of its significance, the *C. reinhardtii* ∼110 Mb haploid genome was among the earliest eukaryotic genomes to be sequenced (Grossman et al., 2003; Merchant et al., 2007), and both the genome assembly and annotation are continuously being developed and improved upon (Blaby et al., 2014). Despite its quality and extensive application, *C. reinhardtii* currently meets the “phylogenetically isolated” definition. The closest confirmed relatives of *C. reinhardtii* with available genome assemblies belong to the clade of multicellular algae that includes *Volvox carteri*, the *Tetrabaenaceae–Goniaceae–Volvocaceae*, or TGV clade. Collectively, *C. reinhardtii* and the TGV clade are part of the highly diverse order Volvocales, and the more taxonomically limited clades *Reinhardtinia* and core-*Reinhardtinia* (Nakada et al., 2008, 2016). Although these species are regularly considered close relatives, multicellularity likely originated in the TGV clade over 200 million years ago (Herron et al., 2009), and *C. reinhardtii* and *V. carteri* are more divergent from one another than human is to chicken (Prochnik et al., 2010).

Without a comparative genomics framework, the wider application of *C. reinhardtii* as a model system is impeded. While this broadly applies to the general functional annotation of the genome as outlined above (e.g. refinement of gene models and annotation of CEs), it is particularly relevant to the field of molecular evolution. Although the evolutionary biology of *C. reinhardtii* has not been widely studied, the species has several features that have attracted recent attention to its application in this field. Its haploid state, high genetic diversity (∼2% genome-wide, Craig et al., 2019) and experimental tractability make it an excellent system to study the fundamental evolutionary processes of mutation (Ness et al., 2015, 2016), recombination (Liu et al., 2018; Hasan and Ness, 2020), and selection (Böndel et al., 2019). However, without genomic resources for closely related species, it is currently impossible to perform several key analyses, such as the comparison of substitution rates at synonymous and non-synonymous sites of protein-coding genes (i.e. calculating dN/dS), and the inference of ancestral states at polymorphic sites (a requirement for several population and quantitative genetics models (Keightley and Jackson, 2018)).

Furthermore, *V. carteri* and its relatives in the TGV clade are extensively used to study the evolution of multicellularity and other major evolutionary transitions (e.g. from isogamy to anisogamy), and five genomes of multicellular species spanning a range of organismal complexities have now been assembled (Prochnik et al., 2010; Hanschen et al., 2016; Featherston et al., 2018; Hamaji et al., 2018). These studies have often included analyses of gene family evolution, reporting expansions in families thought to be functionally related to multicellularity. While these analyses have undoubtedly made important contributions, they are nonetheless limited in their phylogenetic robustness, as *C. reinhardtii* is the only unicellular relative within hundreds of millions of years available for comparison. Thus, the availability of annotated genomes for unicellular relatives of *C. reinhardtii* will also serve as an important resource towards reconstructing the ancestral core-*Reinhardtinia* gene content, potentially offering new clues into the major evolutionary transitions that have occurred in this lineage.

Here, we present highly contiguous and well-annotated genome assemblies for the two closest known relatives of *C. reinhardtii*, namely *Chlamydomonas incerta* (Göttingen culture collection, SAG 7.73) and *Chlamydomonas schloesseri* (Culture Collection of Algae and Protozoa, CCAP 11/173), and a more distantly related unicellular species, *Edaphochlamys debaryana* (CCAP 11/70). Via comparison to the genomes of *C. reinhardtii* and the TGV clade species, we present foundational knowledge of *Chlamydomonas* comparative genomics, focusing specifically on the conservation of genome architecture between species and the landscape of sequence conservation in *C. reinhardtii*. While forming only one of the initial steps in this process, by providing the first comparative genomics framework for the species we anticipate that these novel resources will greatly aid in the continued development of *C. reinhardtii* as a model organism.

## Results and discussion

### The closest known relatives of *C. reinhardtii*

**Figure 1:**
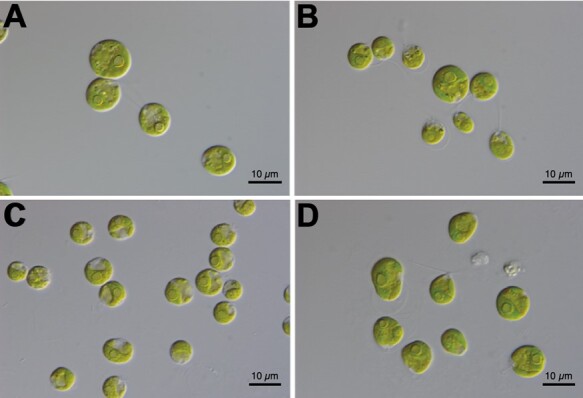
Images of unicellular species. A, *Chlamydomonas reinhardtii*. B, *C. incerta* SAG 7.73. C, *C. schloesseri* SAG 2486 (=CCAP 11/173). D, *E. debaryana* SAG 11.73 (=CCAP 11/70). Scale bars, 20 µm. All images kindly provided by Thomas Pröschold.

Although the genus *Chlamydomonas* consists of several hundred unicellular species, it is highly polyphyletic (Pröschold et al., 2001), and *C. reinhardtii* is more closely related to the multicellular TGV clade than the majority of *Chlamydomonas* species. Given their more conspicuous morphology, the TGV clade contains ∼50 described species (Herron et al., 2009), while the unicellular lineage leading to *C. reinhardtii* includes only two other confirmed species, *C. incerta* and *C. schloesseri* (Pröschold et al., 2005, 2018)*.* As *C. reinhardtii* is the type species of the *Chlamydomonas* genus, these three species collectively comprise the monophyletic genus (Figure 1, A–C), and *Chlamydomonas* will be used specifically to refer to this clade throughout.

*Chlamydomonas incerta* is the closest known relative of *C. reinhardtii*, and a small number of comparative genetics analyses have been performed between the two species (Ferris et al., 1997; Popescu et al., 2006; Smith and Lee, 2008). *Chlamydomonas incerta* is known from only two isolates and we selected the original isolate SAG 7.73 for sequencing. Unfortunately, although *C. incerta* SAG 7.73 is nominally from Cuba, the geographic origin of this isolate is uncertain due to a proposed historical culture replacement with *C. globosa* SAG 81.72 from the Netherlands (Harris et al., 1991). As the direction of replacement is unknown, the strain may be from either location. SAG 7.73 is currently listed as *C. globosa* based on the taxonomic re-assessment of (Nakada et al., 2010), although Pröschold and Darienko (2018) contested this change. We therefore refer to SAG 7.73 as *C. incerta* given its existing use in the genetics literature. *Chlamydomonas schloesseri* was recently described by Pröschold et al. (2018), with the three isolates currently maintained in culture originating from a single site in Kenya. We selected CCAP 11/173 for sequencing.

Beyond *Chlamydomonas*, there are a substantial number of unicellular core-*Reinhardtinia* species with uncertain phylogenetic relationships (i.e. that may be part of the lineage including *Chlamydomonas*, the lineage including the TGV clade, or outgroups to both). Among these, the best studied is *E. debaryana*, which was recently renamed from *Chlamydomonas debaryana* (Pröschold et al., 2018). *Edaphochlamys debaryana* appears to be highly abundant in nature (unlike the three *Chlamydomonas* species), with more than 20 isolates from across the northern hemisphere maintained in culture, suggesting that it would be possible to develop it as a model for studying algal molecular ecology. Draft genomes of the *E. debaryana* isolates NIES-2212 (National Institute for Environmental Studies, Tsukuba, Japan) collected in Japan (Hirashima et al., 2016) and WS7 (also named CC-4515 from the Chlamydomonas Resource Center) from the USA (Nelson et al., 2019) were recently assembled, while we selected CCAP 11/70, collected from the Czech Republic, for sequencing (Figure 1, D). We extracted high-quality genomic DNA from all species according to the protocol provided in Supplemental File S1.

### The genomes of *C. incerta*, *C. schloesseri*, and *E. debaryana*

Using a combination of Pacific Biosciences (PacBio) sequencing for de novo assembly (40–49× coverage, Supplemental Table S1) and Illumina sequencing for error correction (43–86× coverage, Supplemental Table S2), we produced contig-level genome assemblies for *C. incerta*, *C. schloesseri*, and *E. debaryana* (see Supplemental File S2 for details). All three assemblies were highly contiguous, with N50s (the shortest contig length from a series of contigs covering 50% of the genome) of 1.6 Mb (*C. incerta*), 1.2 Mb (*C. schloesseri*), and 0.73 Mb (*E. debaryana*), and L50s (the smallest number of contigs whose total length equals 50% of the genome) of 24, 30, and 56 contigs, respectively (Table 1). Benchmarking universal single-copy orthologs (BUSCO) genome mode scores also supported a high-level of assembly completeness, with the percentage of universal chlorophyte single-copy orthologs identified in each genome ranging from 95.9% to 98.1%. These metrics compared favorably to the best existing core-*Reinhardtinia* (Table 1) and Volvocales assemblies (Supplemental Table S3). Although the *C. reinhardtii* and *V. carteri* assemblies have greater scaffold-level N50s than our three new assemblies, they are both considerably more fragmented at the contig level, with N50s of 215 and 85 kb, respectively. While such a difference is not surprising, given our application of long-read sequencing, it nonetheless demonstrates that these important model genomes can be substantially improved by additional sequencing efforts. At the contig-level, the N50 values of the three new assemblies also exceeded the N50s of the assemblies of the colonial algae *Gonium pectorale* (Hanschen et al., 2016), *Yamagishiella unicocca*, and *Eudorina* sp. *2016-703-Eu-15* (hereafter *Eudorina* sp.), with the final two assemblies also being PacBio-based (Hamaji et al., 2018).

**Table 1:** Genome assembly metrics for eight high-quality core-*Reinhardtinia* genome assemblies

Species	*Chlamydomonas reinhardtii* v5	*Chlamydomonas incerta*	*Chlamydomonas schloesseri*	*Edaphochlamys debaryana*	*Gonium pectorale*	*Yamagishiella unicocca*	*Eudorina. sp. 2016-703-Eu-15*	*Volvox carteri* v2
Assembly level	chromosome	contig	contig	contig	scaffold	contig	scaffold	scaffold
Assembly size (Mb)	111.10	129.24	130.20	142.14	148.81	134.23	184.03	131.16
Number of contigs/scaffolds	17^a^	453	457	527	2373	1461	3180	434
N50 (Mb)	7.78	1.58	1.21	0.73	1.27	0.67	0.56	2.60
Contig N50 (Mb)	0.22	1.58	1.21	0.73	0.02	0.67	0.30	0.09
L50	7	24	30	56	30	53	83	15
Contig L50	141	24	30	56	1871	53	155	410
GC (%)	64.1	66.0	64.4	67.1	64.5	61.0	61.4	56.1
TEs and satellites (Mb/%)	15.33/13.80	26.75/20.70	27.48/21.11	20.05/14.11	11.65/7.83	29.57/22.03	46.81 / 25.43	22.22 / 16.94
Simple and low complexity repeats (Mb/%)	8.71/7.84	8.57/7.72	10.19/9.17	6.40/5.76	4.15/3.74	6.55/4.88	15.15 / 8.23	6.45 / 5.80
BUSCO genome mode (complete %/ fragmented %)	96.5/1.7	96.5/1.6	96.1/1.7	94.0/1.9	86.3/4.5	95.9/2.2	94.7 / 2.7	95.9 / 2.4

a 17 chromosomes + 37 unassembled scaffolds.

BUSCO was run using the Chlorophyta odb10 dataset. See Supplemental Table S3 for complete BUSCO results.

Assembled genome sizes varied moderately across the eight species, ranging from 111.1 Mb (*C. reinhardtii*) to 184.0 Mb (*Eudorina* sp.; Table 1). Both *C. incerta* (129.2 Mb) and *C. schloesseri* (130.2 Mb) had consistently larger assemblies than *C. reinhardtii*, while the *E. debaryana* assembly (142.1 Mb) was larger than those of *Y. unicocca* and *V. carteri*. Although additional genome assemblies will be required to fully explore genome size evolution in the core-*Reinhardtinia*, these results suggest that *C. reinhardtii* may have undergone a recent reduction in genome size. Furthermore, while earlier comparisons between multicellular species and *C. reinhardtii* led to the observation that certain metrics of genome complexity (e.g. gene density and intron length, see below) correlate with organismal complexity, these results indicated that genome size, at least for these species, does not. Conversely, as proposed by Hanschen et al. (2016), GC content did appear to decrease with increasing cell number, with genome-wide values ranging from 64.1% to 67.1% for the unicellular species and from 56.1% to 64.5% in the TGV clade (Table 1).

The larger genome sizes of the unicellular species, relative to *C. reinhardtii*, were largely attributed to differences in the content of transposable elements (TEs) and satellite DNA (defined as tandem repeats with monomers >10 bp). We produced repeat libraries for each species by combining manual curation (Supplemental Data Set S1 and Supplemental File S3) with automated repeat identification. For *C. reinhardtii*, we produced an exhaustively curated library that updated all sequences in the existing library available from Repbase (https://www.girinst.org/repbase/) and more than doubled the total number of annotated TEs (267 versus 120 subfamilies), which will be fully described elsewhere. For the three new assemblies, we performed targeted curation of the most abundant TEs in each species, similarly to the annotation performed for the *V. carteri* genome project (Prochnik et al., 2010). All three new assemblies contained greater total amounts (20.1–27.5 Mb) and higher genomic proportions (14.1%–21.1%) of complex repetitive sequence than *C. reinhardtii* (15.3 Mb and 13.8%, respectively; Table 1). As discussed below, the larger genome size of *E. debaryana* was also in part attributed to the substantially higher number of genes present in this species. For all three assemblies, repeat content was relatively consistent across contigs, with the exception of small contigs (<∼100 kb), which exhibited highly variable repeat contents and likely represent fragments of complex regions that have resisted assembly (Supplemental Figure S1). The higher repeat contents of the three assemblies were broadly consistent across TE subclasses (Supplemental Figure S2), although a direct comparison of the TEs present in each genome is complicated by phylogenetic bias. The inclusion of a curated repeat library for *C. reinhardtii* directly contributed to masking and repeat classification in related species, although this effect becomes increasingly negligible as divergence increases and is likely to at least partially explain the lower repeat content and higher proportion of “unknown” classifications observed for *E. debaryana* relative to *C. incerta* and *C. schloesseri* (Table 1 and  Supplemental Figure S2).

Nonetheless, based on manual curation of the most abundant TE families, a qualitative comparison is possible. All curated TEs belonged to subclasses and superfamilies that are present in one or both of *C. reinhardtii* and *V. carteri*, suggesting a largely common repertoire of TEs across the core-*Reinhardtinia*. Alongside more widely recognized elements such as *L1* LINEs (long interspersed nuclear elements) and *Gypsy* LTRs (long terminal repeat retrotransposons), all species contained families of the comparatively obscure *Dualen* LINE elements (Kojima and Fujiwara, 2005), *PAT-like* DIRS (*Dictyostelium* intermediate repeat sequence) elements (Poulter and Butler, 2015), and *Helitron2* rolling-circle elements (Bao and Jurka, 2013). We also identified *Zisupton* and *Kyakuja* DNA transposons, both of which were reported as potentially present in *C. reinhardtii* upon their recent discovery (Böhne et al., 2012; Iyer et al., 2014). Although not the main focus of this study, the annotation of elements from such understudied superfamilies highlights the importance of performing manual TE curation in phylogenetically diverse lineages. Alongside improving our understanding of TE biology, these elements are expected to contribute toward more effective repeat masking/classification and gene model annotation in related species, which will be of increasing importance given the large number of chlorophyte genome projects currently in progress (Blaby-Haas and Merchant, 2019).

### Phylogenomics of the core-*Reinhardtinia* and Volvocales

Due to the low number of available genomes and gene annotations, phylogenetics in the Volvocales has almost exclusively been limited to the study of ribosomal and plastid marker genes. These analyses have successfully delineated several broad clades (e.g. *Reinhardtinia*, *Moewusinia*, *Dunaliellinia*; Nakada et al., 2008), but often yielded inconsistent topologies for more closely related taxa. Utilizing both our own and several recently published genomic resources, we further explored the phylogenomic structure of the core-*Reinhardtinia* and Volvocales. As several genomes currently lack gene annotations, we first used an annotation-free approach, based on the identification of chlorophyte single-copy orthologs with BUSCO (Waterhouse et al., 2018). This data set consisted of 1,624 genes, present in at least 15 out of the 18 included species (12 *Reinhardtinia*, three other Volvocales, and three outgroups from the Sphaeropleales; Supplemental Table S3). For the 11 species with gene annotations (Supplemental Table S4), we produced a second dataset based on orthology clustering for the proteome of each species, which yielded 1,681 single-copy orthologs shared by all species. For both datasets, we then performed maximum-likelihood (ML) analyses using IQ-TREE (Nguyen et al., 2015). Analyses were performed on both concatenated protein alignments (producing a species-tree) and individual alignments of each ortholog (producing gene trees), which were then summarized as a species-tree using ASTRAL-III (Zhang et al., 2018).

**Figure 2:**
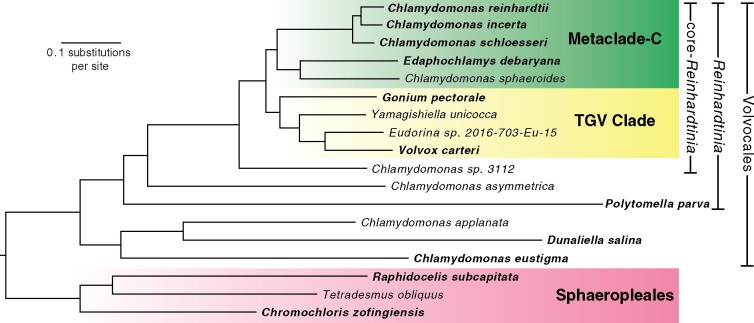
ML phylogeny of 15 Volvocales species and three outgroups. The phylogeny was inferred using the LG+F+R6 model and a concatenated protein alignment of 1,624 chlorophyte BUSCO genes. All ultrafast bootstrap values ≥99%. Species in bold have gene model annotations and were included in the OrthoFinder-based phylogenies (Supplemental Figure S3, B and C). Phylogeny was rooted on the three Sphaeropleales species (highlighted in pink).

All four of the resulting phylogenies exhibited entirely congruent topologies, with near maximal-support values at all nodes (Figure 2 and  Supplemental Figure S3). Rooting the tree on the Sphaeropleales species, we recovered the monophyly of the Volvocales, *Reinhardtinia*, and core-*Reinhardtinia* clades. *Chlamydomonas* was recovered with the expected branching order (Pröschold et al., 2018), as was the monophyly and expected topology of the TGV clade (Nakada et al., 2019). In previous analyses, the most contentious phylogenetic relationships were those of the remaining unicellular core-*Reinhardtinia*, which include *E. debaryana* and the recently published genomes of *Chlamydomonas sphaeroides* (Hirashima et al., 2016) and *Chlamydomonas* sp. 3112 (Nelson et al., 2019). In the most gene-rich analysis to date, *E. debaryana* grouped in a weakly supported clade with *Chlamydomonas* (termed metaclade C), while *C. sphaeroides* grouped with a small number of other unicellular species on the lineage including the TGV clade (Nakada et al., 2019). In our analysis, *E. debaryana* and *C. sphaeroides* were recovered as sister taxa on the lineage that includes *Chlamydomonas*, meeting the prior definition of metaclade C as the sister clade of the TGV clade and its unicellular relatives. Due to its recent discovery, *C.* sp. 3112 has not been included in previous phylogenetic analyses. We classified this species as a member of the core-*Reinhardtinia*, based on sequence similarity of ribosomal and plastid genes, and it is likely a close relative of *Chlamydomonas zebra* (Supplemental Table S5). Given the phylogenetic position as sister to metaclade C and the TGV clade, species such as *C.* sp. 3112 should prove particularly useful in future efforts to reconstruct the ancestral gene content of the core-*Reinhardtinia*.

### Conserved genome architecture and centromeric structure in *Chlamydomonas*

Almost nothing is known about karyotype evolution and the rate of chromosomal rearrangements in *Chlamydomonas* and the core-*Reinhardtinia*. Prochnik et al. (2010) reported that the syntenic genomic segments identified between *C. reinhardtii* and *V. carteri* contained fewer genes than syntenic segments between human and chicken, in part due to a greater number of small inversions disrupting synteny. As the longest contigs in our assemblies were equivalent in length to *C. reinhardtii* chromosome arms (6.4, 4.5, and 4.2 Mb for *C. incerta*, *C. schloesseri*, and *E. debaryana*, respectively), we explored patterns of synteny between the three species and *C. reinhardtii*. We used SynChro (Drillon et al., 2014) to identify syntenic segments, which first uses protein sequence reciprocal best-hits to anchor syntenic segments, before extending segments via the inclusion of homologs that are syntenic but not reciprocal best-hits. All three *Chlamydomonas* genomes were highly syntenous, with 99.5 Mb (89.5%) of the *C. reinhardtii* genome linked to 315 syntenic segments spanning 108.1 Mb (83.6%) of the *C. incerta* genome, and 98.5 Mb (88.6%) of the *C. reinhardtii* genome linked to 409 syntenic segments spanning 108.1 Mb (83.1%) of the *C. schloesseri* genome.

**Figure 3:**
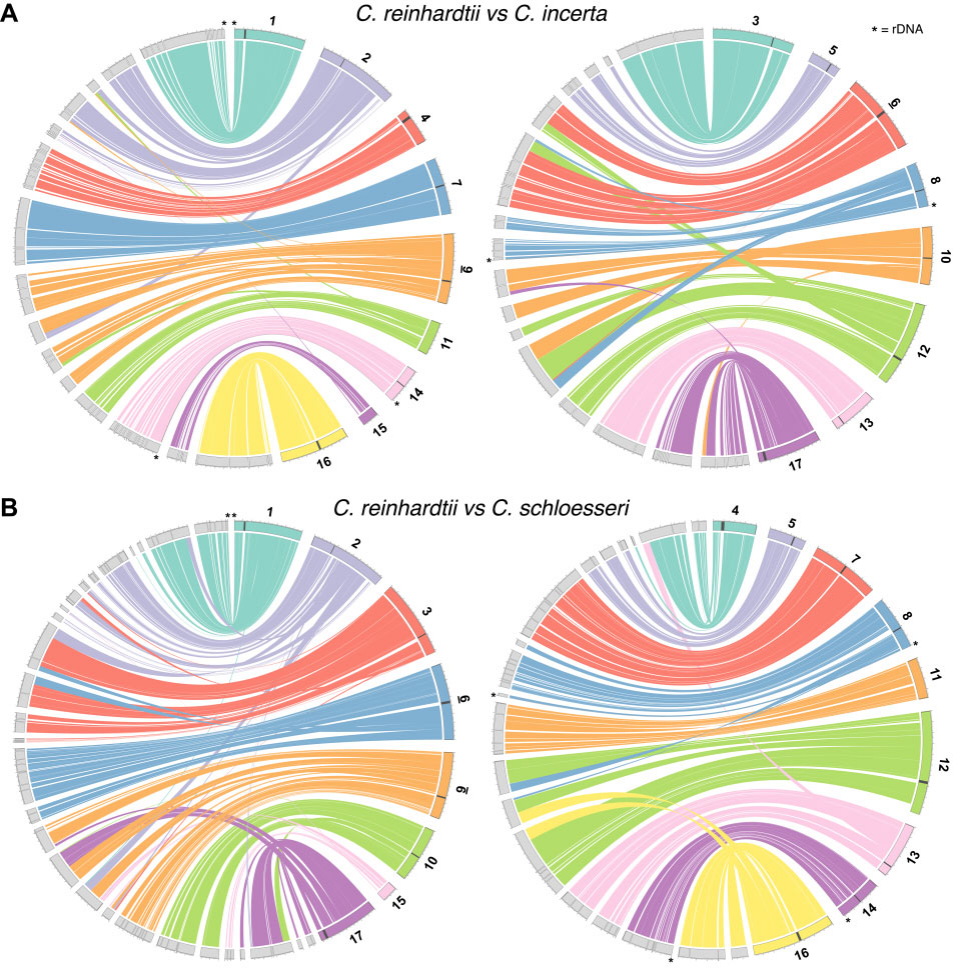
Circos plot (Krzywinski et al., 2009) representation of synteny blocks shared between *C. reinhardtii* and its close relatives. Circos plots between *C. reinhardtii* and *C. incerta* (A) and *C. reinhardtii* and *C. schloesseri* (B). *Chlamydomonas reinhardtii* chromosomes are represented as colored segments and split across the left and right Circos plots, and *C. incerta*/*C. schloesseri* contigs as gray segments. Contigs are arranged and orientated relative to *C. reinhardtii* chromosomes, and adjacent contigs with no signature of rearrangement relative to *C. reinhardtii* are plotted without gaps. Dark gray bands highlight putative *C. reinhardtii* centromeres and asterisks represent rDNA. Note that colors representing specific chromosomes differ between (A) and (B).

Given the high degree of synteny, we ordered and orientated the contigs of *C. incerta* and *C. schloesseri* relative to the *C. reinhardtii* chromosomes (Figure 3). A substantial proportion of the *C. reinhardtii* karyotype appeared to be conserved in *C. incerta*, with six of the 17 chromosomes (1, 3, 4, 7, 14, and 16) showing no evidence of inter-chromosomal rearrangements, and a further three (5, 13, and 15) showing evidence for only minor translocations <150 kb in length (Figure 3, A). Consistent with its greater divergence from *C. reinhardtii*, *C. schloesseri* exhibited such one-to-one conservation for only four chromosomes (5, 7, 11, and 14; Figure 3, B). For both species, patterns of synteny indicated at least one inter-chromosomal rearrangement affecting each of the remaining chromosomes, although it is difficult to comment on the effect of such rearrangements on karyotype without additional scaffolding of contigs. Furthermore, a direct comparison to *C. reinhardtii* chromosomes may overestimate karyotype conservation, due to undetected chromosome fusion/fission events (i.e. if a *C. reinhardtii* chromosome is present as two chromosomes in one of the related species). For both *C. incerta* and *C. schloesseri*, all chromosomes (with the exception of chromosome 15 in the *C. incerta* comparison) contained intra-chromosomal rearrangements relative to *C. reinhardtii*, most of which were small inversions spanning <100 kb (Supplemental Figure S4, A and B). Synteny was far weaker between *C. reinhardtii* and *E. debaryana*, with 58.6 Mb (52.8%) of the *C. reinhardtii* genome linked to 1,975 syntenic segments spanning 64.8 Mb (45.6%) of the *E. debaryana* genome (Supplemental Figure S4, C). Together with the previous assessment of synteny between *C. reinhardtii* and *V. carteri*, these results suggest that karyotype evolution in the core-*Reinhardtinia* is expected to be dynamic, with generally high levels of synteny but a non-negligible rate of inter-chromosomal rearrangements present between closely related species, and likely far greater karyotypic diversity present between more distantly related species.

In view of the high-contiguity and synteny of the assemblies, it was possible to assess features of genome architecture that regularly resist assembly in short-read assemblies. We detected telomeric repeats in all three assemblies, with six *C. incerta* and 19 *C. schloesseri* contigs terminating in the sequence (TTTTAGGG)_*n*_, and 15 *E. debaryana* contigs terminating in (TTTAGGG)_*n*_ (Supplemental Table S6). The *Arabidopsis thaliana-*type sequence (TTTAGGG)_*n*_ is ancestral to green algae and was previously confirmed as the telomeric repeat in *E. debaryana*, while the derived *Chlamydomonas-*type sequence (TTTTAGGG)_*n*_ was found in both *C. reinhardtii* and *V. carteri* (Fulnečková et al., 2012). Given the phylogenetic relationships presented above (Figure 2), the observed telomeric repeats implied either two independent transitions to the derived sequence, or a reversion to the ancestral sequence in the lineage that includes *E. debaryana*, providing further evidence for the relatively frequent transitions that have produced extensive variation in telomere composition in green algae and land plants (Peska and Garcia, 2020). Ribosomal DNA repeats (rDNA) were assembled as part of three larger contigs in both *C. incerta* and *C. schloesseri*, but were found only as fragmented contigs entirely consisting of rDNA in *E. debaryana*. Although poorly assembled in *C. reinhardtii*, the rDNA arrays are located at subtelomeric locations on chromosomes 1, 8, and 14, where cumulatively they are estimated to be present in 250–400 tandem copies (Howell, 1972; Marco and Rochaix, 1980). The assembled *C. incerta* and *C. schloesseri* rDNA arrays (which are not complete and are present in five tandem copies at the most) were entirely syntenous with those of *C. reinhardtii*, suggesting conservation of subtelomeric rDNA organization in *Chlamydomonas* (Figure 3). The subtelomeric regions of *C. reinhardtii* and the three newly assembled genomes were recently described by Chaux-Jukic et al., (2021).

**Figure 4:**
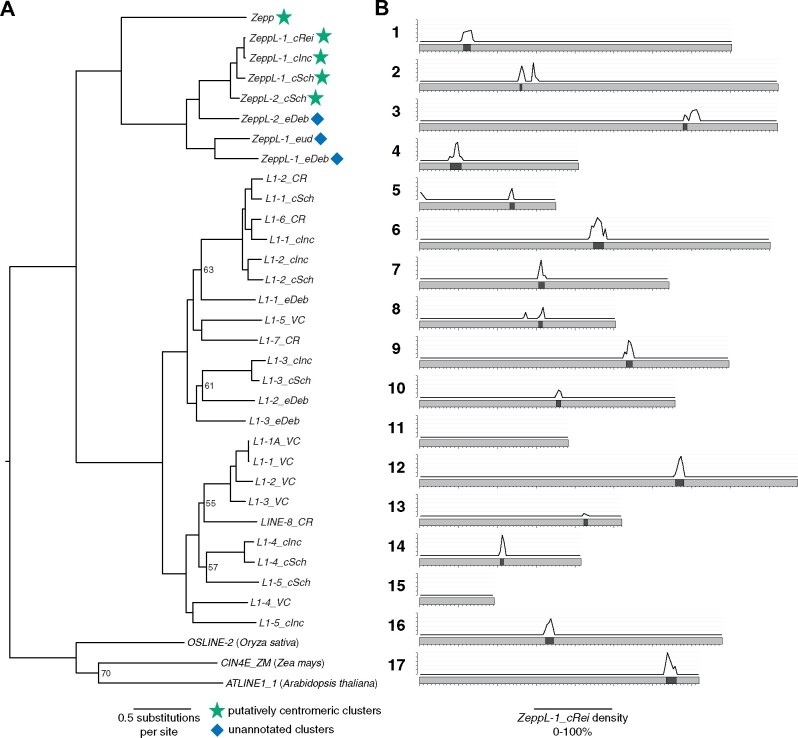
Phylogenetic relationship and centromeric clustering of *Zepp*-like elements. A, ML phylogeny of chlorophyte *L1* LINE elements inferred using the LG+F+R6 model and alignment of endonuclease and reverse transcriptase protein domains. Bootstrap values ≤70% are shown. Phylogeny is rooted on plant *L1* elements. Species are provided by the element name suffix, as follows: *CR/cRei* = *C. reinhardtii*; *VC* = *V. carteri*; *cInc* = *C. incerta*; *cSch* = *C. schloesseri*; *eDeb* = *E. debaryana eud* = *Eudorina* sp. 2016-703-Eu-15. B, Density (0%–100%) of *ZeppL-1_cRei* in 50 kb windows across *C. reinhardtii* chromosomes. Dark bands represent putative centromeres, *x*-axis ticks represent 100-kb increments and *y*-axis ticks represent 20% increments. Plot produced using karyoploteR (Gel and Serra, 2017). Note that *ZeppL-1_cRei* is a synonym of the Repbase element *L1-1_CR* (see Supplemental Data Set S1).

Finally, we were able to assess the composition and potential synteny of centromeres in *Chlamydomonas*. The centromeric locations of 15 out of the 17 *C. reinhardtii* chromosomes were recently mapped by Lin et al. (2018), who observed that these regions were characterized by multiple copies of genes encoding reverse transcriptase domains. Upon inspection of these regions, we found that the majority of these genes are encoded by copies of the *L1* LINE element *L1-1_CR*. Although these regions are currently not sufficiently well assembled to conclusively define the structure of centromeric repeats, *L1-1_CR* was present in multiple copies at all 15 putatively mapped centromeres and appeared to be the major centromeric component (with chromosome-specific contributions from other TEs, especially *Dualen* LINE elements) (Supplemental Table S7 and Supplemental Figure S5, A). Remarkably, phylogenetic analysis of all curated *L1* elements from green algae indicated that *L1-1_CR* is more closely related to the *Zepp* elements of the polar unicellular green alga *Coccomyxa subellipsoidea* than to any other *L1* elements annotated in *C. reinhardtii* (Figure 4, A). The divergence of the classes Trebouxiophyceae (to which *C. subellipsoidea* belongs) and Chlorophyceae (to which *C. reinhardtii* belongs) occurred in the early Neoproterozoic era (i.e. 700–1,000 million years ago) (Del Cortona et al., 2020), suggesting that *L1-1_CR* has been evolving independently from all other *C. reinhardtii L1* elements for more than half a billion years. *Zepp* elements are thought to constitute the centromeres in *C. subellipsoidea*, where they are strictly present as one cluster per chromosome (Blanc et al., 2012). The clustering pattern of *Zepp* arises from a nested insertion mechanism that targets existing copies, creating tandem arrays consisting mostly of the 3′-end of the elements (due to frequent 5′-truncations upon insertion; (Higashiyama et al., 1997)). Chromosome-specific clustering of *L1-1_CR* was also evident in *C. reinhardtii*, with highly localized clusters observed at all 15 putative mapped centromeres (Figure 4, B). The double peaks in *L1-1_CR* density seen on chromosomes 2, 3, and 8, and the single sub-telomeric cluster present on chromosome 5, are all the result of misassemblies in these highly repetitive regions in the *C. reinhardtii* version 5 assembly and will be fully described elsewhere. Thus, outside the putative centromeres, *L1-1_CR* appears to be entirely absent from the *C. reinhardtii* genome. To distinguish the updated annotation of *L1-1_CR* in our repeat library (Supplemental Data Set S1 and Supplemental File S3) from the original Repbase version, we propose the name *ZeppL-1_cRei*, where *ZeppL* stands for *Zepp*-like.

Every putative centromeric location in *C. reinhardtii* coincided with breaks in syntenic segments and the termination of contigs in *C. incerta* and *C. schloesseri* (Figure 3), suggesting that these regions are also likely to be repetitive in both species. The phylogenetic analysis revealed the presence of one and two *ZeppL-1_cRei* homologs in *C. incerta* and *C. schloesseri*, respectively (Figure 4, A). Of the 30 syntenous contig ends associated with the 15 *C. reinhardtii* centromeres, 28 contigs in both species contained a *ZeppL* element within their final 20 kb (Supplemental Figure S5, B and C). Genome-wide, *ZeppL* elements exhibited a similarly localized clustering to that observed in *C. reinhardtii* (Supplemental Figure S6, A and B). Thus, both the location and composition of the *C. reinhardtii* centromeres are likely conserved in *C. incerta* and *C. schloesseri*. We also identified two families of *ZeppL* elements in the *E. debaryana* genome and one family of *ZeppL* elements in the *Eudorina* sp. genome, although we did not find any evidence for *ZeppL* elements in either *Y. unicocca* or *V. carteri*. Given the lack of synteny between *C. reinhardtii* and *E. debaryana*, it was not possible to assign putatively centromeric contigs. Nonetheless, we observed highly localized genomic clustering of *ZeppL* elements for both *E. debaryana* and *Eudorina* sp. (Supplemental Figure S6, C and D), suggesting that these elements may play a similar role as in *Chlamydomonas*.

As sequencing technologies advance, it is becoming increasingly clear that TEs, alongside satellite DNA, contribute substantially to centromeric sequence in many species (Chang et al., 2019; Fang et al., 2020). In light of the evolutionary distance between *C. subellipsoidea* and *Chlamydomonas*, it is tempting to predict that *ZeppL* elements may be present at the centromeres of many other green algal species. However, it is unlikely that centromeres are conserved between species from the Trebouxiophyceae and Chlorophyceae. First, centromeric repeats in the Chlorophyceae species *Chromochloris zofingiensis* consist of entirely unrelated *Copia* LTR elements (Roth et al., 2017). Second, the apparent absence of *ZeppL* elements from *Y. unicocca* and *V. carteri* suggests that these elements are not required for centromere formation in these species. Instead, it is possible that the propensity for *Zepp* and *ZeppL* elements to form clusters may play a role in their recruitment as centromeric sequences, which is likely to have happened independently in *C. subellipsoidea* and *Chlamydomonas*. As more highly contiguous chlorophyte assemblies become available, it will be important to search these genomes for* ZeppL* clusters to assess whether these elements can be used more generally as centromeric markers.

### Gene and gene family evolution in the core-*Reinhardtinia*

We performed gene annotation for each species using 7.4–8.2 Gb of stranded transcriptome deep sequencing (RNA-seq) data (Supplemental Table S8). Protein mode BUSCO scores supported a high level of annotation completeness across all three species (97.0%–98.1% of chlorophyte genes present), although there was an increase in the proportion of fragmented genes (4.0%–5.9%) relative to genome mode scores (Table 2). *Chlamydomonas incerta* and *C. schloesseri* had gene counts comparable to those in *C. reinhardtii*, although at lower gene densities as a result of their larger genomes. With 19,228 genes, the *E. debaryana* genome contained substantially more genes than any other currently annotated core-*Reinhardtinia* species. As reported by Hanschen et al. (2016), several metrics appeared to correlate with organismal complexity. Relative to the unicellular species, gene density was lower, and median intergenic and intron lengths were longer, in *G. pectorale* and *V. carteri*. Presumably, this is at least partly due to an increase in the amount of regulatory sequence in these genomes, although this remains to be explored.

**Table 2:** Gene annotation metrics for core-*Reinhardtinia* species

Species	*Chlamydomonas reinhardtii* v5.6^a^	*Chlamydomonas incerta*	*Chlamydomonas schloesseri*	*Edaphochlamys debaryana*	*Gonium pectorale*	*Volvox* *carteri* v2.1
Number of genes	16,656	16,350	15,571	19,228	16,290	14,247
Number of transcripts	18,311	16,957	16,268	20,450	16,290	16,075
Gene coverage (Mb/%)	91.22/82.10	94.42/73.06	94.29/73.42	103.13/72.55	65.04/43.71	84.00/64.04
UTR coverage (Mb/%)	17.32/15.59	14.51/11.22	12.02/9.23	14.68/9.31	0/0	15.15/11.55
Mean intron number	7.81	8.58	7.67	9.31	6.15	6.73
Median intron length (bp)	229	225	244	198	310	343
Median intergenic distance (bp)	134	341	408	555	2372	905
BUSCO protein mode (complete %/fragmented %)	96.1/2.3	91.1/5.9	94.7/3.0	94.1/4.0	81.5/12.9	94.7/2.0

a *Chlamydomonas reinhardtii* annotation is based on a customized repeat-filtered version of the v5.6 annotation (see the “Materials and methods” section).

Intron metrics are based only on introns within coding sequence, to avoid differences caused by the quality of UTR annotation. BUSCO was run using the Chlorophyta odb10 dataset. See Supplemental Table S4 for complete BUSCO results.

Across all species, both mean intron lengths (discussed below) and intron numbers per gene were very high for genomes in this size range. For the unicellular species, the mean number of introns per gene coding sequence ranged from 7.7 to 9.3, with slightly lower mean counts in *G. pectorale* (6.2) and *V. carteri* (6.7). These numbers were more comparable to those of vertebrates such as human (8.5) than to other model organisms with similar genomes sizes, such as *Caenorhabditis elegans* (5.1), *Drosophila melanogaster* (3.0), and *A. thaliana* (4.1). Modeling of intron evolution across the breadth of eukaryota has predicted that a major expansion of introns occurred early in chlorophyte evolution, and that high intron densities have since been maintained in certain lineages by a balance between intron loss and gain (Csuros et al., 2011). It has been hypothesized that the relative roles of DNA double-strand break repair pathways play a major role in the dynamics of intron gain and loss, as homologous recombination (HR) is thought to cause intron deletion, while nonhomologous end-joining (NHEJ) may result in both intron gain and loss (Farlow et al., 2011). It is worth noting that HR occurs at an extremely low rate in *C. reinhardtii* (Zorin et al., 2005), and if this is shared across the core-*Reinhardtinia* it may contribute to the maintenance of such high intron numbers. Alternatively, introns may be maintained by other forces, such as selection. Notably, high rates of NHEJ have also recently been linked to high GC content in prokaryotes (Weissman et al., 2019), and it may be the case that double-strand break repair is generally an important and underappreciated force in *Chlamydomonas* genome evolution.

**Figure 5:**
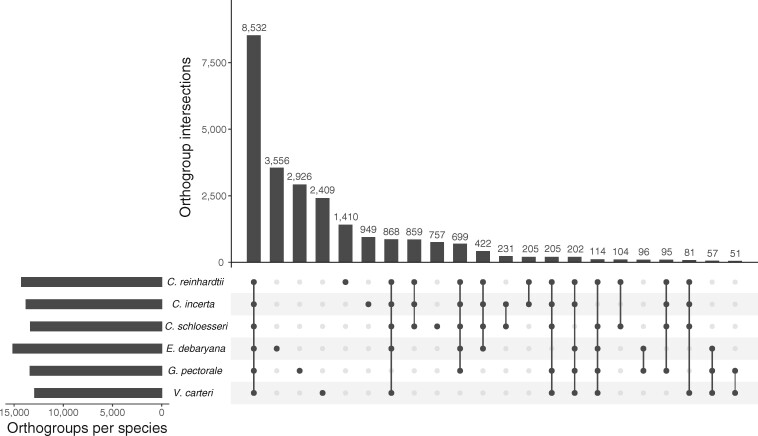
Upset plot (Lex et al., 2014) representing the intersection of orthogroups between six core-*Reinhardtinia* species. Numbers above bars represent the number of orthogroups shared by a given intersection of species.

To explore gene family evolution in the core-*Reinhardtinia*, we performed orthology clustering using the six available high-quality gene annotations (98,342 total protein-coding genes), which resulted in the delineation of 13,728 orthogroups containing 86,446 genes (Figure 5). Most orthogroups (8,532) were shared across all species, with the second most abundant category (excluding genes unique to a single species) being those present in all species except *G. pectorale* (868 orthogroups). Given the lower BUSCO score observed for *G. pectorale* (Table 2), we hypothesize that a proportion of these orthogroups are also universal to core-*Reinhardtinia* species. The next most abundant category was the 859 orthogroups present only in *Chlamydomonas*. Unfortunately, essentially nothing is known about the biology and ecology of *C. incerta* and *C. schloesseri*, and even for *C. reinhardtii* we have a minimal understanding of its biology in natural environments (Sasso et al., 2018; Craig et al., 2019). Nonetheless, more than 30% of the *Chlamydomonas*-specific orthogroups were associated with at least one functional domain (Supplemental Table S9). The most common association was with protein kinase domains (50 orthogroups), followed by other relatively common domains in *C. reinhardtii* including peptidase M11/gametolysin (14 orthogroups). *Chlamydomonas reinhardtii* is known to encode a large kinome relative to other unicellular green algae (Wheeler et al., 2008), with 575 *C. reinhardtii* genes encoding proteins annotated with protein kinase domains in our current analysis, 86 of which were present in *Chlamydomonas*-specific orthogroups. With 51 genes, the most gene-rich *Chlamydomonas*-specific orthogroup represented the *NCL* (*Nuclear Control of Chloroplast gene expression* [*NCC*]-*Like*) gene family. These genes encode RNA binding proteins of unknown function, are entirely absent from *V. carteri*, and are undergoing a rapid diversification in *C. reinhardtii* via recurrent gene duplication that has formed a cluster of at least 32 genes on chromosome 15 (Boulouis et al., 2015). Both *C. incerta* and *C. schloesseri* contained six genes in the *NCL* orthogroup, all of which were syntenous with chromosome 15 in *C. reinhardtii*. It therefore appears that although the *NCL* genes evolved in the common ancestor of *Chlamydomonas*, most of the diversification is specific to *C. reinhardtii* itself and attempts to uncover the evolutionary driver of the rapid expansion should focus on biological differences between *C. reinhardtii* and its closest relatives. In contrast to *Chlamydomonas*, only 51 orthogroups were unique to the two multicellular species. This number may be an underestimate due to the relative incompleteness of the *G. pectorale* annotation, and it will be important to re-visit this analysis as more annotations become available (e.g. for *Y. unicocca* and *Eudorina* sp.). Nonetheless, the availability of the three new high-quality annotations for unicellular species will provide a strong comparative framework to explore the relative roles of gene family birth versus expansions in existing gene families in the transition to multicellularity.

Finally, we explored the contribution of gene family expansions to the high gene count of *E. debaryana*. The *E. debaryana* genome contained more species-specific genes (3,556) than any other species; however, this figure was not substantially higher than the unassigned gene counts for *G. pectorale* and *V. carteri* (Figure 5). We quantified *E. debaryana* gene family expansion and contraction by calculating per orthogroup log_2_-transformed ratios of the *E. debaryana* gene count and the mean gene count for the other species. Arbitrarily defining an expansion as a log_2_-transformed ratio >1 (i.e. a given orthogroup containing more than twice as many *E. debaryana* genes than the mean of the other species) and a contraction as a ratio <–1, we identified *E. debaryana*-specific expansions in 294 orthogroups and contractions in 112. With 16 genes in *E. debaryana* compared to at most one in the other five species, the most expanded orthogroup contained genes encoding scavenger receptor cysteine-rich (SRCR) and C-type lectin (CTL) domains (Supplemental Table S10). SRCR and CTL domains have roles in innate immunity in animals and the presence of >30 genes encoding SRCR and/or CTL domains in *C. reinhardtii*, which may have roles in immunity or other processes such as chemoreception, was a surprising finding from the genome project (Wheeler et al., 2008). Other orthogroups exhibiting the most extreme expansions were associated with HIT and MYND-type zinc fingers, polyketide cyclase SnoaL-like domains, protein kinase domains and pherophorins (Supplemental Table S10), although in all cases the *C. reinhardtii* and *V. carteri* genes present in these orthogroups were not annotated with specific functions. Furthermore, more than 100 of the expanded orthogroups were not associated with any functional domains at all. Only ∼50% of *C. reinhardtii* genes are annotated with domains and only ∼10% are formally annotated with primary gene symbols (Blaby and Blaby-Haas, 2017). Further exploring the relationships between gene content and the biological differences of *C. reinhardtii* and its close relatives may be a powerful approach to functionally characterize additional genes, especially those that are unique to specific clades such as the Volvocales or core-*Reinhardtinia*.

### Evolution of the mating type locus in *Chlamydomonas*

Across core-*Reinhardtinia* species, sex is determined by a haploid mating-type locus (*MT*) with two alleles, termed *MT*^+^ or female, and *MT*^–^ or male, in isogamous and anisogamous species. The *C. reinhardtii MT* locus is located on chromosome 6, spanning >400 kb and consisting of three domains, the T (telomere-proximal), R (rearranged), and C (centromere-proximal) domains. While both the T and C domains exhibit high synteny between *MT* alleles, the R domain contains the only *MT*-specific genes (Ferris and Goodenough, 1997) and harbors substantial structural variation, featuring several inversions and rearrangements (Ferris et al., 2002; De Hoff et al., 2013). Crossover events are suppressed across the *MT* locus, although genetic differentiation between gametologs is reduced as a result of widespread gene conversion (De Hoff et al., 2013; Hasan et al., 2019). Comparative analyses of *MT*^+^/female and *MT*^–^/male haplotypes between *C. reinhardtii* and TGV clade species have revealed highly dynamic *MT* locus evolution, with extensive gene turnover and structural variation resulting in a complex and discontinuous evolutionary history of haplotype reformation (Ferris et al., 2010; Hamaji et al., 2016b, 2018). This is most strikingly illustrated by the male R domains of *V. carteri* and *Eudorina sp.*, the former being ∼1.1 Mb in length and relatively repeat-rich, while the latter is just 7 kb and contains only three genes (Hamaji et al., 2018). Only one *MT*-specific gene is common to all species, *MINUS DOMINANCE* (*MID*), which determines *MT*^–^/male gametic differentiation (Ferris and Goodenough, 1997).

To explore whether *MT* locus evolution is similarly dynamic between the more closely related *Chlamydomonas* species, we used a reciprocal best-hit approach to identify *C. reinhardtii MT* orthologs in *C. incerta* and *C. schloesseri*. The sequenced isolates of both species were inferred to be *MT*^–^ based on the presence of *MID*, as was previously reported for *C. incerta* (Ferris et al., 1997). Orthologs of *MT locus, region d* (*MTD1*), the second and only other *MT*^–^-specific gene in *C. reinhardtii*, were also identified in both species. Although we were able to map the entire *C. reinhardtii MT*^–^ haplotype to single contigs in both the *C. incerta* and *C. schloesseri* assemblies, it is important to state that it is currently impossible to define the R domain boundaries for either species without sequencing their *MT*^+^ alleles. Unfortunately, it is currently unknown if any of the one (*C. incerta*) or two (*C. schloesseri*) other isolates are *MT*^+^. In addition, as no isolate from either species has been successfully crossed, it is not even known if they are sexually viable (Pröschold et al., 2005). Furthermore, as sexual reproduction has not been observed for either species, it cannot definitively be stated that they are heterothallic or even possess *MT* loci at all, as *MID* orthologs are present and required for sexual development in homothallic species in the TGV clade (Hamaji et al., 2013; Yamamoto et al., 2017). To test this possibility, we explored patterns of synonymous codon usage in both species. Assuming that patterns of *MT* locus recombination are similar to those in *C. reinhardtii*, we would expect *MID* (and possibly also *MTD1*) to exhibit little evidence of selection acting on codon usage if *C. incerta* and *C. schloesseri* are heterothallic, due to low selection efficacy caused by the absence of recombination (both crossovers and gene conversion). Indeed, *MID* in *C. incerta* was previously shown to have the lowest codon adaptation index (CAI) among a dataset of 67 genes (Popescu et al., 2006). We quantified codon adaptation for all genes using the index of translation elongation (*I*_TE_), a metric that takes mutation bias into account (unlike CAI) but can otherwise be interpreted analogously (Xia, 2015). In both species, *MID* was within the lowest 2% of genes for *I*_TE_ genome-wide and had the lowest *I*_TE_ of any gene present on the contigs syntenous to the *C. reinhardtii MT* locus (Supplemental Figure S7). *MTD1* also exhibited low *I*_TE_ in *C. schloesseri* (lowest ∼9% of genes), although the reduction in *C. incerta* was less pronounced (lowest ∼23%). These results support the presence of *MT* loci in both species, although it is possible that *MTD1* may not be *MT*^–^-specific in *C. incerta* (as is found in *Y. unicocca* and *Eudorina* sp., Hamaji et al., 2018). We therefore proceed with this assumption, although confirming this will require the sequencing of the other existing isolates or new isolates in the future. Finally, we also determined the sequenced isolate of *E. debaryana* to be *MT*^–^ based on the identification of *MID*, although we did not explore *MT* locus evolution further, given the evolutionary distance to *C. reinhardtii*. Unlike *C. incerta* and *C. schloesseri*, heterothallic mating pairs of *E. debaryana* are in culture, and a future comprehensive study of the *MT* locus in the species is therefore possible.

**Figure 6:**
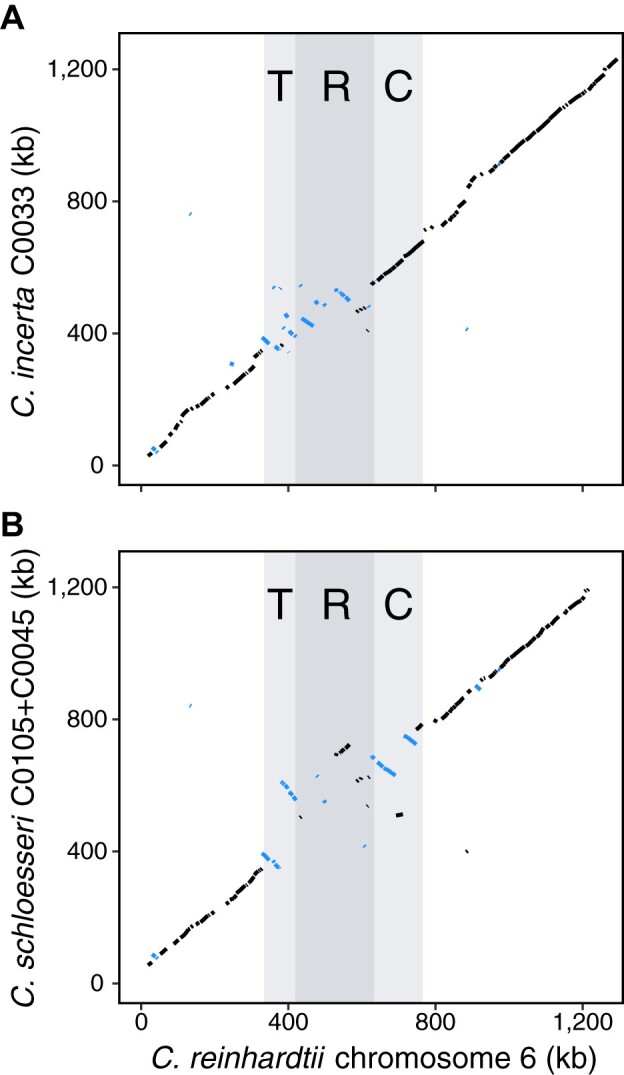
Synteny representation across the *C. reinhardtii MT*^–^ haplotype and inferred *MT*^–^ haplotypes in *C. incerta* and *C. schloesseri*. Shown is the synteny between the *C. reinhardtii* genes across the *MT*^–^ haplotype and flanking autosomal sequence and (A) inferred *C. incerta MT*^–^ haplotype and flanking sequence genes (contig C0033), or (B) inferred *C. schloesseri MT*^–^ haplotype and flanking sequence genes (contig C0045). The T, R, and C domains of the *C. reinhardtii MT*^–^ are highlighted. Genes with inverted orientations are shown in blue. Note that for *C. schloesseri*, the region syntenous to the *C. reinhardtii MT* is entirely on contig C0045, but C0105 was appended to C0045 to show the genes syntenous with the most telomere-proximal region of *C. reinhardtii* chromosome 6.

In *C. incerta*, gene order was entirely syntenic across the C domain, with the exception of the zygote-specific gene *MT0828* (Cre06.g254350), which did not yield a hit anywhere in the genome. Conversely, both T and R domain genes have undergone several rearrangements and inversions relative to *C. reinhardtii MT*^–^ (Figure 6, A). Furthermore, the T domain genes *SIGNAL PEPTIDE PEPTIDASE 3* (*SPP3*) and *HALOACID DEHALOGENASE-LIKE HYDROLASE1* (*HDH1*, currently annotated as *PGP6*) were present on separate contigs in *C. incerta* and did not appear to be linked to *MT*^–^ (Supplemental Table S11). Synteny otherwise continued well into the adjacent autosomal sequence, in line with the genome-wide patterns of synteny described above. We observed even less synteny between *C. reinhardtii* and *C. schloesseri MT*^–^ genes, with both the T and C domains showing two large inversions each (Figure 6, B). However, gene order in the surrounding autosomal sequence was also largely collinear. As in *C. incerta*, *SPP3* was located elsewhere in the *C. schloesseri* assembly, suggesting a relatively recent translocation to the T domain in *C. reinhardtii*. The T domain gene *97782* (Cre06.g251750) was also located on a different contig, while the genes *MT0796* (Cre06.g254175), *MT0828*, and *182389* (Cre06.g252050) did not yield hits anywhere in the *C. schloesseri* genome. Finally, we found no hits for the *MT*^+^-specific genes *FUSION 1* (*FUS1*) and *MT locus*, *region a* (*MTA1*) in either species, suggesting that these genes (assuming they exist) are also expected to be *MT*^+^-specific.

The lack of collinearity relative to the *C. reinhardtii* T domain may be indicative of an extended R domain in these species, especially in *C. schloesseri*, where we observe multiple rearrangements in all three domains. We did not, however, observe dramatic variation in *MT* size; whereas *C. reinhardtii MT*^–^ is ∼422 kb, if *NICOTINAMIDE-REQUIRING 7* (*NIC7*) and *MATERNAL 3* (*MAT3*) are taken as the boundaries of the locus (De Hoff et al., 2013), *C. incerta MT*^–^ is ∼329 kb and *C. schloesseri MT*^–^ is ∼438 kb. In all, while we do find evidence of *MT*^–^ haplotype reformation within *Chlamydomonas*, this is mostly limited to rearrangements, with far less gene turnover and *MT* locus size variation than has been observed between more distantly related core-*Reinhardtinia* species. While *MT* locus evolution has previously been explored in the context of transitions from unicellularity to multicellularity and isogamy to anisogamy, our data suggest that *MT* haplotype reformation is still expected to occur between closely related isogamous species, albeit at a reduced scale.

### Alignability and estimation of neutral divergence

**Figure 7:**
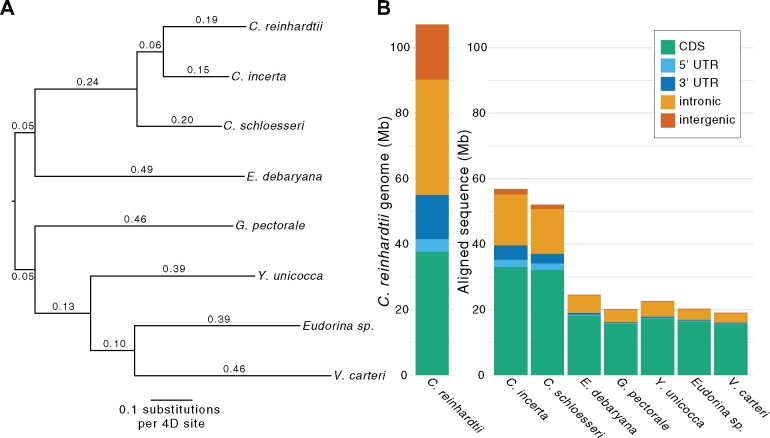
Putatively neutral divergence and alignability across the core-*Reinhardtinia*. A, Estimates of putatively neutral divergence under the GTR model, based on the topology of Figure 2 and 1,552,562 *C. reinhardtii* 4D sites extracted from the Cactus WGA. B, A representation of the *C. reinhardtii* genome by site class, and the number of aligned sites per *C. reinhardtii* site class for each other species in the Cactus WGA.

In order to facilitate the identification of CEs and the assessment of current *C. reinhardtii* gene models, we produced an eight-species core-*Reinhardtinia* WGA using Cactus (Armstrong et al., 2019). Based on the alignment of *C. reinhardtii* four-fold degenerate (4D) sites extracted from the WGA, we estimated putatively neutral branch lengths across the topology connecting the eight species under the general time reversible (GTR) substitution model (Figure 7, A). We estimated the divergence between *C. reinhardtii* and *C. incerta*, and *C. reinhardtii* and *C. schloesseri*, to be 34% and 45%, respectively. Divergence between *C. reinhardtii* and *E. debaryana* was estimated as 98%, while all four TGV clade species were saturated relative to *C. reinhardtii* (i.e. on average, each 4D site is expected to have experienced more than one substitution). To put these estimates within a more recognizable context, divergence across *Chlamydomonas* is approximately on the scale of human-rodent divergence (Lindblad-Toh et al., 2011), while divergence between *Chlamydomonas* and the TGV clade is roughly equivalent to that between mammals and sauropsids (birds and reptiles), which diverged ∼320 million years ago (Alföldi et al., 2011). Our estimates corroborated a previous estimate of synonymous divergence between *C. reinhardtii* and *C. incerta* of 37% (Popescu et al., 2006) and were broadly in line with the divergence time estimate of ∼230 million years between the TGV clade and their unicellular ancestors (Herron et al., 2009). It is important to note that we have likely underestimated neutral divergence, as 4D sites are unlikely to be evolving neutrally due to selection acting on codon usage, which has been shown to reduce divergence between *C. reinhardtii* and *C. incerta* (Popescu et al., 2006).

As expected, genome-wide alignability (the proportion of bases aligned between *C. reinhardtii* and a given species in the WGA) decreased substantially with increasing divergence, with 53.0% of the *C. reinhardtii* genome aligning to *C. incerta*, 48.6% to *C. schloesseri*, and on average only 19.9% to the remaining five species (Figure 7, B). The majority of *C. reinhardtii* coding sequence (CDS) was alignable within *Chlamydomonas* (87.7% and 85.5% to *C. incerta* and *C. schloesseri*, respectively), indicating that it will be possible to perform molecular evolutionary analyses (e.g. calculating dN/dS) between the three species. CDSs also constituted the majority of the aligned sequence to the other five species, comprising on average 78.3% of the aligned bases despite forming only 35.2% of the *C. reinhardtii* genome. By contrast, far less nonexonic sequence was alignable, especially beyond *Chlamydomonas*. Substantial proportions of intronic bases were aligned to *C. incerta* (44.1%) and *C. schloesseri* (38.8%), with on average 11.3% aligned to the other five species. Less than 10% of intergenic sequence aligned to any one species, and on average less than 1% aligned to non-*Chlamydomonas* species. Distributions of intergenic tract lengths across the core-*Reinhardtinia* were highly skewed (Supplemental Figure S8), so that in *C. reinhardtii* tracts shorter than 250 bp constituted 63.5% of tracts but just 5.5% of total intergenic sequence. The sequence content of tracts >250 bp was highly repetitive (total repeat content 63.4%), while tracts <250 bp were relatively free of repeats (4.3% repeat content) and as a result were far more alignable to *C. incerta* and *C. schloesseri* (40.8% and 32.0% of bases aligned, respectively). This observation suggests that, at least for introns and short intergenic tracts, it is feasible to explore the landscape of nonexonic evolutionary constraint, primarily utilizing alignment data from *Chlamydomonas*, supplemented by what is likely the alignment of only the most conserved sites at greater evolutionary distances.

### False positive and missing genes in *C. reinhardtii*

**Figure 8:**
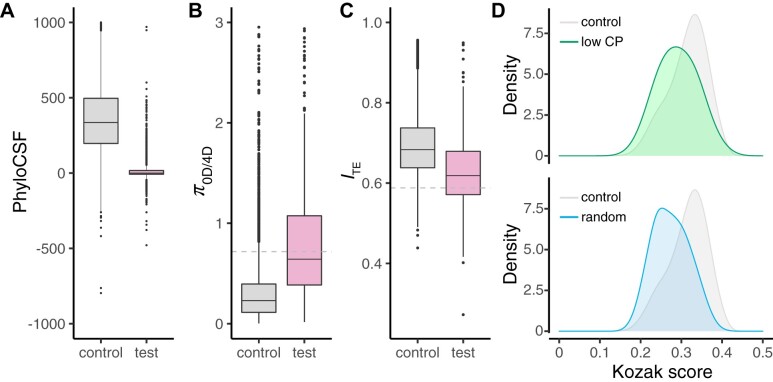
Coding potential analyses. A, Boxplot of PhyloCSF scores for control and test set genes. B, Boxplot of the ratio of genetic diversity at 0D and 4D sites (*π*_0D/4D_) for control and test set of genes. Gray dashed line represents 95th percentile of control gene values. C, Boxplot of codon adaptation, as quantified by *I*_TE_ for control and test set genes. Gray dashed line represents fifth percentile of control gene values. D, Density plot of Kozak scores, quantified as the per gene agreement of the start codon sequence context to that of the *C. reinhardtii* Kozak consensus sequence. Low CP (i.e. low coding potential), the 250 test set genes that failed all three coding potential analyses; control, the opposite half of the control set to that used to produce the Kozak consensus sequence (see the “Materials and methods” section); random, 10,000 sequences generated based on an average GC content of 64.1%

One of the major successes of comparative genomics has been the refinement of gene annotations. Many approaches that utilize WGAs rely on the ability to distinguish between protein-coding and noncoding sequence, and programs such as PhyloCSF (Lin et al., 2011) quantify coding potential by assessing candidate alignments for evolutionary signatures characteristic of CDS, such as higher synonymous and lower nonsynonymous divergence. Using our new resources, we first attempted to assess the prevalence of false-positive genes in the current *C. reinhardtii* v5.6 annotation. Prior to this, we checked the v5.6 annotation against our new *C. reinhardtii* TE library, and surprisingly found that 1,022 genes showed >30% overlap between CDS and TEs. The distribution of CDS-TE overlap across genes was extremely bimodal, with ∼99% of genes exhibiting either <20% or >80% overlap (Supplemental Figure S9), cleanly distinguishing a set of genes encoded by TEs that are currently included in the v5.6 annotation. Taking into account genes overlapping simple and other repeats (e.g. rDNA), we filtered out 1,085 genes (∼6% of v5.6 genes), which is reflected in Table 2 and has been used throughout for all comparative analyses (see the “Materials and methods” section). There are several implications of this result that will be fully detailed in a future manuscript. We divided the remaining 16,656 genes into a “control” set that contained all genes with at least one core-*Reinhardtinia* ortholog and/or encoding a functional domain (15,365 genes), and a “test” set that failed both conditions (1,291 genes). We ran PhyloCSF on alignments of CDS extracted from the WGA, producing a per gene score (with more positive scores indicating a higher coding potential). The score distributions for the control and test gene sets were strikingly different, with a median score of 359.9 for the control set and 0 for the test set (with scores of 0 in almost all cases representing a complete lack of alignment; Figure 8, A). In full, 865 test set genes (∼67%) scored <1, while the same was true for 598 control set genes (∼4%). The positive scores and likely true positive status for approximately one-third of the test set may be explained by the orthologs of these genes being absent from the annotations for the aligned species (as PhyloCSF is not reliant on gene annotations from outgroup species). Alternatively, many of these genes may be fast evolving at the protein-level, thus escaping orthology clustering. Of the remaining test set genes, caution must be taken in designating false positive status, since this subset may include genes unique to *C. reinhardtii* (i.e. orphan genes or recent gene duplications). There is also expected to be a false positive rate associated with PhyloCSF caused by misalignment or a lack of power (i.e. for genes where CDS does not align across several of the species in the WGA), as demonstrated by the ∼4% of genes scoring <1 in the control set.

We therefore performed two further analyses to more accurately delineate a set of false positive gene models. First, for each gene we calculated the ratio of genetic diversity (*π*) at zero-fold degenerate (0D) and 4D sites based on whole-genome re-sequencing data from 17 *C. reinhardtii* field isolates from Quebec (Craig et al., 2019). As would be expected under an assumption of purifying selection, median *π*_0D/4D_ for the control set was 0.230 and <2% of genes had a ratio >1 (Figure 8, B). Conversely, median *π*_0D/4D_ for the test set was 0.665 and ∼30% of genes had a ratio >1. Taking the 95th percentile of control *π*_0D/4D_ (0.717) as a cut-off, 823 test set genes exceeded this threshold (or *π*_0D/4D_ could not be calculated at all), 626 of which also had a PhyloCSF score <1. Second, we quantified codon adaptation for each gene using *I*_TE_, under the assumption that false positive genes would be expected to deviate from the overall codon usage bias of *C. reinhardtii*. Median *I*_TE_ for the control set was 0.683, dropping to 0.619 for the test set (Figure 8, C). Taking the fifth percentile of control *I*_TE_ (0.588) as a cut-off, 430 test set genes were below this threshold, 345 of which had a phyloCSF score <1. Considering the three analyses together, 250 test set genes (∼19%) had a PhyloCSF score <1 and had π_0D/4D_ and *I*_TE_ values exceeding the control set thresholds, while 721 (∼56%) genes had a PhyloCSF score <1 and exceeded one but not both thresholds. We designate these genes as low coding potential, with the exact number of false positive gene models in the v5.6 annotation likely falling somewhere between the sets of 250 and 721 genes.

There are several biological reasons why genuine protein-coding genes may have outlying values in the above analyses. For example, genes evolving under positive selection (e.g. immune system genes) may exhibit an excess of nonsynonymous substitutions or variants, affecting both the PhyloCSF score and *π*_0D/4D_. As with *MID* detailed above, genes evolving in low recombination regions may be expected to have sub-optimal codon usage. Nonetheless, there are several additional features of the genes designated as low coding potential that support their likely status as false positive models. Focusing on the set of 250 genes, their open-reading frames (ORFs) were considerably shorter (mean 372.2 bp) and consisted of fewer exons (mean 2.1 exons) than the remaining genes (means 2293.2 bp and 8.9 exons). GC content at third codon positions was substantially lower (mean 65.5%) relative to the remaining genes (mean 81.9%) and was only marginally higher than the genome-wide GC content (64.1%) that would be expected in random sequence. Genetic diversity of high impact sites (start codons, 0D sites in stop codons, and splice junctions) was an order of magnitude higher (0.0177) relative to the remaining genes (0.000983) and was of the same order as genetic diversity genome-wide (see below), indicating that many of the ORFs from the low coding potential gene set are disrupted by variants at the population-level. Finally, the putative start codons of low coding potential genes generally lacked strong Kozak sequences, suggesting that they possess unfavorable sequence context for translational initiation. Following Cross (2015), we calculated a 'Kozak” score' for each gene based on the agreement between the *C. reinhardtii* Kozak consensus sequence and the information content in bits per site for the five bases up and downstream of each start codon. The distribution of Kozak scores for the low coding potential genes more closely resembled random sequence (Figure 8, D) and did not produce a recognizable Kozak consensus sequence (Supplemental Figure S10).

Given the complexity and probabilistic nature of gene prediction, the presence of several hundred likely false positives is not unexpected, with even the most developed annotations such as human containing a non-negligible number of dubious gene models (Abascal et al., 2018). This is especially true given the high GC content of *C. reinhardtii*, since the length of ORFs expected by chance increases with GC content due to decreasing stop codon frequency (Pohl et al., 2012). The mean ORF length of the low coding potential set (∼124 codons) was not substantially longer than the 100 codons that is often used as a statistically robust threshold. Indeed, as there are genuine protein coding genes of <100 amino acids and several functionally characterized lncRNAs that contain spurious ORFs of >100 codons, a clean designation of coding and noncoding sequence based on ORF length is not possible in any case (Housman and Ulitsky, 2016). Assuming that they are expressed, it is possible that many of these gene models are in fact lncRNAs, which have not yet been thoroughly characterized in *C. reinhardtii*. The one study that annotated lncRNAs in the species filtered any transcripts that overlapped existing gene annotations (Li et al., 2016), which despite being a logical approach may have resulted in many lncRNAs being discarded. Given the compactness of the *C. reinhardtii* genome, an alternative possibility is that many of the false positive genes are in fact spurious ORFs within the untranslated regions (UTRs) of neighboring genes. Further approaches such as long-read RNA sequencing will be required to distinguish between such hypotheses.

Finally, we attempted to identify *C. reinhardtii* genes absent from the current v5.6 genome annotation using a similar comparative approach. We performed de novo gene prediction, which yielded 433 novel gene models. We reduced this dataset to 142 high-confidence missing genes based on the gene models either having a PhyloCSF score >100 or a syntenic homology in one or both of *C. incerta* and *C. schloesseri* (based on the SynChro approach described above). Supporting their validity, 37 or the 142 genes contained a functional domain. Furthermore, 35 had significant Basic Local Alignment Search Tool for proteins (BLASTP) hits (>95% sequence similarity, ≥80% query protein length) to *C. reinhardtii* proteins from annotation v4.3 (Supplemental Table S12) and likely represent models that were lost during the transition from v4 to v5 of the genome. This is a known issue with the current annotation, and our rediscovered gene set includes fundamental genes such as *psbW* (encoding the W protein of photosystem II), which had been previously recorded as missing (Blaby and Blaby-Haas, 2017). Notably, 25 of the 142 genes were recently found to be part of polycistronic transcripts (Gallaher et al. 2021).

### The genomic landscape of sequence conservation in *C. reinhardtii*

Based on the WGA, we identified 265,006 CEs spanning 33.8 Mb or 31.5% of the *C. reinhardtii* genome. The majority of CE sites overlapped CDSs (70.6%), with the remaining sites overlapping 5′ UTRs (2.9%), 3′ UTRs (4.4%), introns (20.0%), and intergenic sites (2.0%; Table 3). Relative to the site class categories themselves, 63.1% of CDSs, 24.8% of 5′ UTRs, 11.0% of 3′ UTRs, and 19.2% of intronic sites overlapped with CEs. Only 4.1% of intergenic sites overlapped with CEs, although splitting intergenic tracts into those <250 bp (short tracts) and >250 bp (long tracts), increased the overlap with CEs more appreciably along short tract sites (14.1%). As would be predicted given the expectation that CEs contain functional sequences, genetic diversity was 39.5% lower for CEs (0.0134) than non-CE bases (0.0220), a result that was relatively consistent across site classes, with the exception of long intergenic tracts (Table 3). It is important to state that the CEs we have identified here contain a proportion of nonconstrained sites. While this is always to be expected to some extent (e.g. CDS is generally included in CEs despite the presence of synonymous sites), our CE dataset (with a mean length of 128 bp) should be cautiously interpreted as regions containing elevated proportions of constrained sites.

**Table 3:** Overlap between CEs and *C. reinhardtii* genomic site classes

Site class	CE overlap (Mb)	Proportion of CE bases (%)	Proportion of site class (%)	Genetic diversity all sites (*π*)	Genetic diversity CE sites (*π*)	Genetic diversity non-CE sites (*π*)
CDS	23.85	70.64	63.10	0.0144	0.0112	0.0204
5′-UTR	0.97	2.86	24.76	0.0189	0.0138	0.0208
3′-UTR	1.48	4.38	10.97	0.0205	0.0151	0.0213
Intronic	6.76	20.01	19.15	0.0248	0.0216	0.0256
Intergenic <250 bp	0.13	0.38	14.07	0.0229	0.0194	0.0235
Intergenic ≥250 bp	0.56	1.65	3.55	0.0137	0.0134	0.0138

Due to the compactness of the *C. reinhardtii* genome (82.1% genic, median intergenic tract length 134 bp), a high proportion of regulatory sequence is expected to be concentrated in UTRs and intergenic sequences immediately upstream of genes (i.e. promoter regions). Relatively little is known about the genome-wide distribution of regulatory elements in *C. reinhardtii*, although analyses based on motif modeling have identified putative cis-regulatory elements in these regions (Castruita et al., 2011; Ding et al., 2012; Hamaji et al., 2016a). Presumably, many CEs overlapping UTRs and promoter regions harbor regulatory elements, and the CEs we have identified may be used in future studies to validate potential functional motifs (i.e. by assessing whether predicted motifs overlap with CEs). However, since CE lengths are generally considerably longer than the expected length for regulatory elements, genomes for additional close relatives of *C. reinhardtii* (assuming such species exist) would be required to achieve sufficient power to directly identify novel regulatory elements.

**Figure 9:**
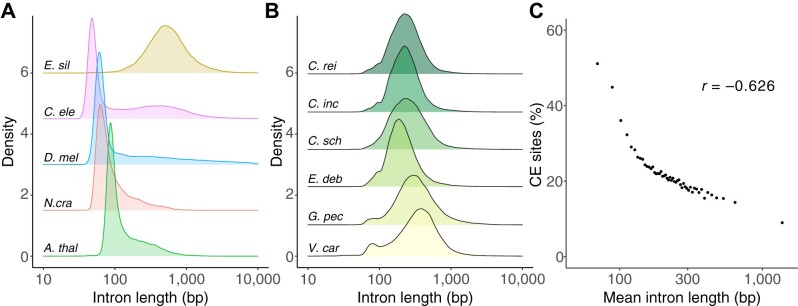
Intron lengths and overlap with CEs. A, Intron length distributions for five model organisms (*C. ele* = *C. elegans*, *D. mel* = *D. melanogaster*, *N. cra* = *Neurospora crassa*, *A. thal* = *A. thaliana*, *E. sil* = *Ectocarpus siliculosus*). The brown alga *E. siliculosus* is included as an example of an atypical distribution similar to that seen in the core-*Reinhardtinia*. B, Intron length distributions for six core-*Reinhardtinia* species (*C. rei* = *C. reinhardtii*, *C. inc* = *C. incerta*, *C. sch* = *C. schloesseri*, *E. deb* = *Edaphochlamys debaryana*, *G. pec* = *G. pectorale*, *V. car* = *V. carteri*). C, Correlation between mean intron length per bin and the proportion of sites overlapped by CEs. Introns were ordered by length and separated into 50 bins, so that each bin contained the same number of introns.

All six annotated core-*Reinhardtinia* species contained conspicuously long introns (median lengths 198–343 bp, Table 2). As reported previously for *C. reinhardtii* (Merchant et al., 2007), the distribution of intron lengths for core-*Reinhardtinia* species lacked the typical peak in intron lengths at 60–110 bp that is present in several model organisms with similarly compact genomes (Figure 9, A and B). In *D. melanogaster*, short introns (<80 bp) appear to largely consist of neutrally evolving sequence, while longer introns that form the tail of the length distribution contain sequences evolving under evolutionary constraint (Halligan and Keightley, 2006). To explore the relationship between intron length and sequence conservation in *C. reinhardtii*, we ordered introns by length and divided them into 50 bins, so that each bin contained an approximately equal number (∼2,667) of introns. Mean intron length per bin was significantly negatively correlated with the proportion of sites overlapped by CEs (Pearson’s *r =* –0.626, *P* < 0.01; Figure 9, C). This observation was particularly pronounced for introns <100 bp (∼5% of introns), for which 48.1% of sites were overlapped by CEs, compared to 18.5% for longer introns. Therefore, it appears that in a reverse of the situation found in *D. melanogaster*, the minority of introns in *C. reinhardtii* are short and contain a high proportion of conserved sites, while the majority of introns are longer and are expected to contain a higher proportion of sites evolving under little constraint. The tight peak in the distribution of intron lengths, combined with the lack of sequence constraint in *D. melanogaster* short introns, led Halligan and Keightley (2006) to hypothesize that intron length was under selection, but not the intronic sequence itself, and that introns had essentially evolved to be as short as possible. It is possible that *C. reinhardtii* introns are similarly evolving under selection to be bounded within certain length constraints, although the selective advantage of maintaining intron lengths substantially longer than the minimum remains unknown. Given that atypical intron length distributions are common to all core-*Reinhardtinia* species, whatever mechanism is driving intron length is likely evolutionarily ancient.

There are several reasons why intronic sites might be evolving under evolutionary constraint. First, alternative splicing (AS) can result in the incorporation of either the entire intron (i.e. intron retention, IR) or part of an intron (alternative acceptor or donor splice sites) into the mature mRNA. IR is the most common form of AS in *C. reinhardtii* (∼30% of AS events) and occurs significantly more frequently in shorter genes (median = 181 bp; Raj-Kumar et al., 2017). However, AS in the species has not yet been extensively characterized and only ∼1% of introns are currently annotated as alternatively retained. Second, many microRNA and small nuclear RNA (snoRNA) genes have been identified within introns of protein-coding genes (Chen et al., 2008; ValLi et al., 2016). Perhaps most importantly, many introns are expected to contain regulatory sequences. This is especially true for introns within the first 1-kb downstream of the transcription start site, which for many genes have strong regulatory effects on gene expression (Rose, 2018). The addition of a specific first intron to transgenes in *C. reinhardtii* has also been shown to substantially increase their expression (Baier et al., 2018). Short introns <100 bp were found to be first introns approximately four-times more frequently (44.6%) than longer introns (10.3%) (Supplemental Figure S11, A) and were also significantly more likely to occur closer to the transcription start site (mean intron position relative to transcript length for introns <100 bp = 24.2% and introns >100 bp = 39.5%; independent samples *t* test *t*=−54.0, *P* < 0.01; Supplemental Figure S11, B). Caution should be taken not to overinterpret any differences between short and long introns, as the relationship between intron length and the proportion of CE sites (Figure 9, C) is likely driven by shorter introns containing fewer non-constrained sites relative to longer introns (as opposed to shorter introns containing more constrained sites overall). Nonetheless, the enrichment of shorter introns at the start of genes may be worthy of further attention for any possible functional implications on gene regulation.

Finally, we identified 5,611 ultraconserved elements (UCEs) spanning 356.0 kb of the *C. reinhardtii* genome, defined as sequences ≥50 bp and exhibiting 100% sequence conservation across the three *Chlamydomonas* species. A subset of just 55 UCEs showed ≥95% sequence conservation across all eight species, indicating that hardly any sequence is expected to be conserved at this level across the core-*Reinhardtinia*. The majority of UCE sites (96.0%) overlapped CDSs, indicating constraint at both nonsynonymous and synonymous sites. There are several reasons why synonymous sites may be subject to such strong constraint, including interactions with RNA binding proteins, the presence of exonic regulatory elements, or selection for optimal codon usage. Noticeably, 15 of the 55 core-*Reinhardtinia* UCEs overlapped ribosomal protein genes, which are often used as standards for identifying optimal codons given their extremely high gene expression levels (Sharp and Li, 1987), and several of the other genes overlapped by UCEs are also expected to be very highly expressed (e.g. elongation factors) (Supplemental Table S13). Although considered to be a very weak evolutionary force, this raises the possibility that coordinated selection for optimal codons across the core-*Reinhardtinia* may be a driver of extreme sequence conservation. Alternatively, many of the UCEs may be the result of RNA binding constraints, as certain ribosomal proteins may bind and autoregulate their own mRNA (Müller-McNicoll et al., 2019). UCEs have proven to be excellent phylogenetic markers across several taxa (Faircloth et al., 2012, 2015). Given the lack of nuclear markers and the current difficulty in determining phylogenetic relationships in the core-*Reinhardtinia*, the 55 deeply CEs may potentially be used to provide additional phylogenetic resolution.

## Conclusion

With the assembly of highly contiguous and well-annotated genomes for three of *C. reinhardtii*’s unicellular relatives, we have presented a thorough nucleotide-level comparative genomics framework for this important emerging model. These resources are expected to enable the continued development of *C. reinhardtii* as a model system for molecular evolution. Furthermore, by providing deeper knowledge into the gene content and genomic architecture of unicellular core-*Reinhardtinia* species, these resources are also expected to advance our understanding of the genomic changes that have occurred during the transition to multicellularity in the TGV clade.

These genome assemblies only now raise *C. reinhardtii* to a standard comparable to that achieved for many other model organisms ten or more years ago. Many of the analyses we have performed would be greatly enhanced by the inclusion of additional *Chlamydomonas* species; however, addressing this is more a question of taxonomy than sequencing effort. *Chlamydomonas* is in this regard somewhat analogous to the past situation for *Caenorhabditis*, where only very recent advances in ecological knowledge have led to a rapid increase in the number of sampled species and sequenced genomes (Stevens et al., 2019). We hope that this study will encourage the *Chlamydomonas* community to increase sampling efforts for new species, fully realizing the power of comparative genomics analyses in the species.

## Materials and methods

### Nucleic acid extraction and sequencing

Algal isolates were obtained from the Culture Collection of Algae at Göttingen University (SAG) or CCAP, Scottish Marine Institute culture centers, cultured in Bold’s Basal Medium, and where necessary made axenic via serial dilution, plating on agar, and isolation of single algal colonies. We extracted high molecular weight genomic DNA using a customized extension of an existing CTAB/phenol–chloroform protocol (Supplemental File S1). One SMRTbell library (sheared to ∼20 kb, with 15–50 kb size selection) was prepared per species, and each library was sequenced on a single SMRTcell on the PacBio Sequel platform. PacBio library preparation and sequencing were performed by Edinburgh Genomics.

DNA for Illumina sequencing was extracted using a phenol–chloroform protocol (Ness et al., 2012). Across all species, we used a variety of library preparations, read lengths, insert sizes, and sequencing platforms (Supplemental Table S2). Total RNA was extracted from 4-day liquid cultures using Zymo Research TRI Reagent (product ID: R2050) and the Direct-zol RNA Miniprep Plus kit (product ID: R2070) following manufacturer’s instructions. We prepared one stranded RNA-seq library for each species using TruSeq reagents, and sequencing was performed on an Illumina HiSeq X platform (*C. incerta* 150-bp paired-end, *C. schloesseri* and *E. debaryana* 100-bp paired-end). All Illumina sequencing and library preparations were performed by BGI Hong Kong.

### De novo genome assembly

Detailed per-species methods and command line options are detailed in Supplemental File S2. We first identified and removed reads derived from any remaining contaminants by producing taxon-annotated GC-coverage plots with BlobTools v1.0 (Laetsch and Blaxter, 2017a). Assemblies were produced using Canu v1.7.1 (Koren et al., 2017), with three iterative round of error-correction performed with the PacBio reads and the GenomicConsensus module Arrow v2.3.2 (https://github.com/PacificBiosciences/GenomicConsensus). We then used all available Illumina data for each species to perform three iterative rounds of polishing using Pilon v1.22 (Walker et al., 2014). Complete plastid assemblies were produced using Circlator (Hunt et al., 2015). Mitochondrial assemblies were produced by Smith and Craig (2021).

### Annotation of genes and repetitive elements

A preliminary repeat library was produced for each species with RepeatModeler v1.0.11 (http://www.repeatmasker.org/RepeatModeler/). Repeat models with homology to *C. reinhardtii* v5.6 and/or *V. carteri* v2.1 transcripts (e-values <10-3, MEGABLAST (Camacho et al., 2009)) were filtered out. The genomic abundance of each repeat model was estimated by providing RepeatMasker v4.0.9 (http://www.repeatmasker.org) with the filtered RepeatModeler output as a custom library, and any TEs with a cumulative total >100 kb were selected for manual curation, following Suh et al. (2014). Briefly, multiple copies of a given TE were retrieved by querying the appropriate reference genome using MEGABLAST, before each copy was extended at both flanks and aligned using MAFFT v7.245 (Katoh and Standley, 2013). Alignments were then manually inspected, consensus sequences were created, and TE families were classified following Wicker et al. (2007) and Kapitonov and Jurka (2008). This procedure was also performed exhaustively for *C. reinhardtii* (i.e. curating all de novo repeat models and existing Repbase models regardless of genomic abundance), which will be described in detail elsewhere. Final repeat libraries were made by combining the RepeatModeler output for a given species with all novel curated TEs and *V. carteri* repeats from Repbase (Supplemental Data Set S1 and Supplemental File S3). TEs and satellites were soft-masked by providing RepeatMasker with the above libraries. In line with the most recent *C. reinhardtii* annotation (Blaby et al., 2014), low-complexity and simple repeats were not masked, as the high GC-content of genuine CDS can result in excessive masking.

Adapters and low-quality bases were trimmed from each RNA-seq dataset using Trimmomatic v0.38 (Bolger et al., 2014) with the parameters optimized by Macmanes (2014). Trimmed reads were then mapped to repeat-masked assemblies with the two-pass mode of STAR v2.6.1a (Dobin et al., 2013). Gene annotation was performed with BRAKER v2.1.2 (Hoff et al., 2016, 2019), an automated pipeline that combines the gene prediction tools Genemark-ET (Lomsadze et al., 2014) and AUGUSTUS (Stanke et al., 2006, 2008). Read pairs mapping to the forward and reverse strands were extracted using samtools v1.9 (Li et al., 2009) and passed as individual BAM files to BRAKER, which was run with the “–UTR=on” and “–stranded=+,-” flags to perform UTR annotation. Resulting gene models were filtered for genes with internal stop codons, protein sequences <30 amino acids, or CDS overlapped by ≥30% TEs/satellites or ≥70% low-complexity/simple repeats. Proteins were functionally annotated via upload to the Phycocosm algal genomics portal (https://phycocosm.jgi.doe.gov).

### Phylogenomic analyses

Genome and gene annotations for all available *Reinhardtinia* species and selected outgroups (Supplemental Tables S3, S4) were accessed from either Phytozome v12 (if available) or the National Center for Biotechnological Information (NCBI). For annotation-based analyses, protein clustering analysis was performed with OrthoFinder v2.2.7 (Emms and Kelly, 2015), using the longest isoform for each gene, the modified BLASTP options “-seq yes, -soft_masking true, -use_sw_tback” (following Moreno-Hagelsieb and Latimer (2008)) and the default inflation value of 1.5. Protein sequences from orthogroups containing a single gene in all 11 included species (i.e. putative single copy orthologs) were aligned with MAFFT and trimmed for regions of low-quality alignment using trimAl v1.4.rev15 (“-automated1”; Capella-Gutiérrez et al., 2009). A ML species-tree was produced using concatenated gene alignments with IQ-TREE v1.6.9 (Nguyen et al., 2015), run with ModelFinder (“-m MFP”; Kalyaanamoorthy et al., 2017) and ultrafast bootstrapping (“-bb 1000”; Hoang et al., 2018). ASTRAL-III v5.6.3 (Zhang et al., 2018) was used to produce an alternative species-tree from individual gene-trees, which were themselves produced for each aligned single copy ortholog using IQ-TREE as described above, with any branches with bootstrap support <10% contracted as recommended.

Annotation-free phylogenies were produced from a dataset of single copy orthologous genes identified by BUSCO v3.0.2 (Waterhouse et al., 2018) run in genome mode with the pre-release Chlorophyta odb10 dataset (allowing missing data in up to three species). For each BUSCO gene, proteins were aligned and trimmed, and two-species trees were produced as described above.

### General comparative genomics and synteny analyses

Basic genome assembly metrics were generated using QUAST v5.0.0 (Gurevich et al., 2013). Repeat content was estimated by performing repeat masking on all genomes, as described above (i.e. supplying RepeatMasker with the RepeatModeler output for a given species + manually curated repeats from all species). Assembly completeness was assessed by running BUSCO in genome mode with the Eukaryota odb9 and Chlorophyta odb10 datasets. Each species was run with *C. reinhardtii* (-sp chlamy2011) and *V. carteri* (-sp volvox) AUGUSTUS parameters, and the run with the most complete BUSCO genes was retained.

Syntenic segments were identified between *C. reinhardtii* and the three new genomes described here using SynChro (Drillon et al., 2014) with a block stringency value (delta) of 2. To create the input file for *C. reinhardtii*, we combined the repeat-filtered v5.6 gene annotation (see below) with the centromere locations for 15 of the 17 chromosomes, as defined by Lin et al. (2018). The resulting synteny blocks were used to check the *C. incerta* and *C. schloesseri* genomes for misassemblies, by manually inspecting breakpoints between synteny blocks on a given contig that resulted in a transition between *C. reinhardtii* chromosomes (see Supplemental File S2). This resulted in four *C. incerta* and two *C. schloesseri* contigs being split due to likely misassembly.

A ML phylogeny of *L1* LINE elements was produced from the endonuclease and reverse transcriptase domains (i.e. ORF2) of all available chlorophyte *L1* elements. Protein sequences were aligned, trimmed and analyzed with IQ-TREE as described above. All *C. incerta*, *C. schloesseri* and *E. debaryana* elements were manually curated as part of the annotation of repeats (see above). The *Y. unicocca*, *Eudorina* sp., and *V. carteri* genomes were searched using tBLASTN with the *L1-1_CR* protein sequence as query, and the best hits were manually curated to assess the presence or absence of *ZeppL* elements in these species.

### Gene annotation metrics and gene family evolution

The *C. reinhardtii* v5.6 gene models were manually filtered based on overlap with the new repeat library (see above), which resulted in the removal of 1,085 putative TE/repeat genes. For all species, annotation completeness was assessed by protein mode BUSCO analyses using the Eukaryota odb9 and Chlorophyta odb10 datasets. Gene families were identified using OrthoFinder as described above with the six core-*Reinhardtinia* species with gene annotations (*C. reinhardtii*, *C. incerta*, *C. schloesseri*, *E. debaryana*, *G. pectorale*, and *V. carteri*). Protein sequences for all species were annotated with InterPro domain IDs using InterProScan v5.39-77.0 (Jones et al., 2014). Domain IDs were assigned to orthogroups by KinFin v1.0 (Laetsch and Blaxter, 2017b) if a particular ID was assigned to at least 20% of the genes and present in at least 50% of the species included in the orthogroup.

### Mating-type locus evolution

As the *C. reinhardtii* reference genome is *MT*^+^, we first obtained the *C. reinhardtii MT*^–^ locus and proteins from NCBI (accession GU814015.1) and created a composite chromosome 6 with an *MT*^–^ haplotype. A reciprocal best hit approach with BLASTP was used to identify orthologs, supplemented with tBLASTN queries to search for genes not present in the annotations. To visualize synteny, we used the MCscan pipeline from the JCVI utility libraries v0.9.14 (Tang et al., 2008), which performs nucleotide alignment with LAST (Kiełbasa et al., 2011) to identify orthologs. We applied a C-score of 0.99, which filters LAST hits to only reciprocal best hits, while otherwise retaining default parameters. We manually confirmed that the LAST reciprocal hits were concordant with our BLASTP results.

*I*_TE_ was calculated for each gene using DAMBE7 (Xia, 2018). A reference set of highly expressed genes for each species was delineated by performing correspondence analysis on codon usage, as implemented in CodonW (http://codonw.sourceforge.net) and taking the default 5% of genes from the extreme of axis 1 (after checking that this set was enriched for genes expected to be highly expressed, e.g. histone and ribosomal protein genes). The codon usage for the highly expressed reference genes was then provided to DAMBE7, and *I*_TE_ was calculated for the CDS of each gene using the default option “break 8-fold and 6-fold families into 2”. For both *C. incerta* and *C. schloesseri*, *MID* was annotated by hand, as it was absent from the BRAKER annotations (likely due to its short length and unusual codon usage).

### Whole-genome alignment and divergence estimation

An eight-species core-*Reinhardtinia* WGA was produced using Cactus (Armstrong et al., 2019) with all available high-quality genomes (*C. reinhardtii* v5, *C. incerta*, *C. schloesseri*, *E. debaryana*, *G. pectorale*, *Y. unicocca*, *Eudorina* sp., and *V. carteri* v2). The required guide phylogeny was produced by extracting alignments of 4D sites from single copy orthologs identified by BUSCO (genome mode, Chlorophyta odb10 dataset). Protein sequences derived from 1,543 BUSCO genes present in all eight species were aligned with MAFFT and subsequently back-translated to nucleotide sequences. Sites where the aligned codon in all eight species contained a 4D site were then extracted (250,361 sites), and a guide-phylogeny was produced by supplying the 4D site alignment and topology (extracted from the Volvocales species-tree, see above) to phyloFit (PHAST v1.4; Siepel et al., 2005), which was run with default parameters (i.e. GTR substitution model).

Where available, the R domain of the *MT* locus not included in a given assembly was appended as an additional contig (extracted from the following NCBI accessions: *C. reinhardtii MT*^–^ GU814015.1, *G. pectorale MT*^+^ LC062719.1, *Y. unicocca MT*^–^ LC314413.1, *Eudorina* sp. *MT* male LC314415.1, *V. carteri MT* male GU784916.1). All genomes were soft-masked for repeats as described above, and Cactus was run using the guide-phylogeny, with all genomes set as reference quality. Post-processing was performed by extracting a multiple alignment format (MAF) alignment with *C. reinhardtii* as the reference genome from the resulting hierarchical alignment (HAL) file, using the HAL tools command hal2maf (v2.1; Hickey et al., 2013), with the options –onlyOrthologs and –noAncestors. Paralogous alignments were reduced to one sequence per species by retaining the sequence with the highest similarity to the consensus of the alignment block, using mafDuplicateFilter (mafTools suite v0.1; Earl et al., 2014).

Final estimates of putatively neutral divergence were obtained using a method adopted from Green et al. (2014). For each *C. reinhardtii* protein-coding gene, the alignment of each exon was extracted and concatenated. For the subsequent CDS alignments, a site was considered to be 4D if the codon in *C. reinhardtii* included a 4D site, and all seven other species had a triplet of aligned bases that also included a 4D site at the same position (i.e. the aligned triplet was assumed to be a valid codon, based on its alignment to a *C. reinhardtii* codon). The resulting alignment of 1,552,562 sites was then passed to phyloFit with the species tree, as described above.

### Identification of false positive and missing *C. reinhardtii* genes

PhyloCSF scores were obtained by passing per exon CDS alignments extracted from the WGA to PhyloCSF (Lin et al., 2011), which was run in “omega” mode using the neutral branch length tree obtained from phyloFit. Following Abascal et al. (2018), the per-gene score was taken as the highest scoring exon, since a small section of misalignment or incorrect annotation (which may be localized to a single exon) can cause an overall negative score for the entire CDS of a genuine protein-coding gene. Exon alignments were trimmed to codon boundaries to preserve reading frame and PhyloCSF was only run on exons of at least 45 bp. If no suitable exons were available for a given gene, the score was taken from the entire CDS. Genetic diversity was calculated from re-sequencing data of 17 *C. reinhardtii* field isolates from Quebec (sampled between 1993 and 1994), based on the variant calling and filtering steps described by Craig et al. (2019). *I*_TE_ was calculated for each gene as described above for *C. incerta* and *C. schloesseri*. The Kozak consensus sequence logo for *C. reinhardtii* was determined with WebLogo 3 (Crooks et al., 2004) by providing the 5-bp upstream and downstream of the start codons of a randomly selected half of the control gene set (7,682 genes). Kozak scores were calculated for low coding potential genes, the other half of the control set genes and 10,000 random sequences based on an average genome-wide GC content (64.1%). Following Cross (2015), the score was calculated by summing the per-base bit score from the consensus sequence for each matching base in the query sequence over the 10 sites (i.e. the start codon itself was excluded).

De novo gene annotation was performed on the *C. reinhardtii* v5 genome using BRAKER (without UTR annotation) and all RNA-seq datasets produced by Strenkert et al. (2019). Putatively missing genes were defined as those without any overlap with CDS of v5.6 genes. SynChro was re-run against *C. incerta* and *C. schloesseri* using updated *C. reinhardtii* input files containing the potential new genes. PhyloCSF scores were obtained as above, except scores were taken from entire CDS to ensure only the highest confidence models were retained.

### Identification and analyses of conserved elements

CEs were identified from the eight-species WGA using phastCons (Siepel et al., 2005) with the phyloFit neutral model (described above) and the standard UCSC parameters “–expected-length = 45, –target-coverage = 0.3, –rho = 0.31”. Parameter tuning was attempted, but it proved difficult to achieve a balance between overly long CEs containing too many non-constrained bases at one extreme, and overly fragmented CEs at the other extreme; the standard parameters were found to perform as adequately as others.

*Chlamydomonas reinhardtii* site classes were delineated using the repeat-filtered v5.6 annotation, augmented with the 142 new genes identified (Supplemental File S4). To assess the genomic distribution of conserved bases, site classes were called uniquely in a hierarchical manner, so that if a site was annotated as more than one site class, it was called based on the following hierarchy: CDS, 5′-UTR, 3′-UTR, intronic, intergenic. Overlaps between site classes and CEs were calculated using BEDtools v2.26.0 (Quinlan and Hall, 2010). For analyses of intron length and conservation, all introns were called based on longest isoforms as they appear in the annotation (i.e. no hierarchical calling was performed as described above).

## Accession numbers

Sequence data for genes mentioned in this article can be found at Phytozome or Uniprot under the following accession numbers: *MID* (no Phytozome Cre ID, as the sequenced reference is *MT*^+^ and *MID* is *MT*^–^-specific; Uniprot ID O04101); *MTD1* (no Phytozome Cre ID, as the sequenced reference is *MT*^+^ and *MTD1* is *MT*^–^-specific; Uniprot ID Q945C0); *MT0828* (Cre06.g254350); *SPP3* (Cre06.g251550); *HDH1* (Cre06.g252150); *MT0796* (Cre06.g254175); *97782* (Cre06.g251750); *182389* (Cre06.g252050); *FUS1* (Cre06.g252750); *MTA1* (Cre06.g253000); *NIC7* (Cre06.g251450); *MAT3* (Cre06.g255450); *psbW* (no Phytozome Cre ID; Uniprot ID Q9SPI9). The eight-species core-*Reinhardtinia* Cactus WGA is available from the Edinburgh Datashare repository (doi: https://doi.org/10.7488/ds/2847). All sequencing reads, genome assemblies and gene annotations are available from NCBI under the BioProject PRJNA633871. Assemblies are also available from Phycocosm (https://phycocosm.jgi.doe.gov). Code and bioinformatic pipelines are available at https://github.com/rorycraig337/Chlamydomonas_comparative_genomics, with the exception of the MT analyses which are available at https://github.com/aays/MT_analysis.

## Supplemental data

The following materials are available in the online version of this article.

**Supplemental Figure S1.** Total repeat content per contig for *C. incerta*, *C. schloesseri*, and *E. debaryana*.

**Supplemental Figure S2**. Repeat content per species by repeat subclass.

**Supplemental Figure S3.** Phylogenomic analyses.

**Supplemental Figure S4.** Dotplots representing syntenic genomic segments identified between *C. reinhardtii* and 50 largest contigs of *C. incerta*, *C. schloesseri*, and *E. debaryana.*

**Supplemental Figure S5.** Mean densities of *Zepp*-like *L1* LINE elements per 20 kb windows averaged over relevant chromosomes/contigs.

**Supplemental Figure S6.** Genome-wide density of *Zepp*-like elements.

**Supplemental Figure S7.** Codon adaptation of *minus* mating type genes.

**Supplemental Figure S8.** Distribution of intergenic tract lengths across six core-*Reinhardtinia* species.

**Supplemental Figure S9.** Overlap between coding sequence of *C. reinhardtii* v5.6 genes and manually curated *C. reinhardtii* TEs.

**Supplemental Figure S10.** Kozak consensus sequence logos.

**Supplemental Figure S11.** Relationship between intron lengths and intron locations within genes.

**Supplemental Table S1.** Pacific Biosciences sequencing output. Metrics were calculated after removal of putative contaminant reads.

**Supplemental Table S2.** Illumina genomic DNA sequencing output.

**Supplemental Table S3.** Assembly metrics for genome assemblies of all available *Reinhardtinia* species and selected outgroups.

**Supplemental Table S4.** Gene model annotation metrics for all available *Reinhardtinia* species and selected outgroups.

**Supplemental Table S5.** Best MEGABLAST hits for ribosomal and plastid marker genes of the undescribed species *Chlamydomonas* sp. 3112.

**Supplemental Table S6.** List of contigs terminating in telomeric repeats. Contig start/end refers to the location of the telomeric repeat.

**Supplemental Table S7.** *Chlamydomonas reinhardtii* putative centromeric coordinates and repeat content.

**Supplemental Table S8**. Illumina RNA-seq output.

**Supplemental Table S9.** Orthogroup and InterPro domain annotation for *Chlamydomonas*-specific orthogroups with annotated domains. The *NCL* gene family is represented by OG0000029.

**Supplemental Table S10.** Orthogroup and InterPro domain annotation for *E. debaryana* gene family expansions (log_2_-transformed ratios >1) and contractions (ratios <–1).

**Supplemental Table S11.** Presence-absence of *C. reinhardtii MT*^–^ genes in *C. incerta*, *C. schloesseri* and *E. debaryana.*

**Supplemental Table S12.** New genes identified with significant BLASTP homology to proteins from the *C. reinhardtii* v4.3 annotation.

**Supplemental Table S13.** Core-*Reinhardtinia* UCEs (elements ≥50 bp, 100% conservation within *Chlamydomonas* and ≥95% conservation across eight core-*Reinhardtinia* species).

**Supplemental Data Set S1**. Volvocales-curated TE library.

**Supplemental File S1.** High molecular weight DNA extraction protocol for *Chlamydomonas.*

**Supplemental File S2.** Detailed genome assembly methods.

**Supplemental File S3.** Volvocales-curated TE annotation notes.

**Supplemental File S4.** *Chlamydomonas reinhardtii* v5.6 gene annotation, filtered for TE/repeat genes and with newly identified genes added.

**Supplemental File S5.** OrthoFinder gene clustering used for phylogenomics analyses.

**Supplemental File S6.** Aligned and trimmed OrthoFinder single copy orthologs used for phylogenomics analyses.

**Supplemental File S7.** IQ-TREE phylogeny produced from OrthoFinder single copy orthologs.

**Supplemental File S8.** ASTRAL-III phylogeny produced from OrthoFinder single copy orthologs.

**Supplemental File S9.** Aligned and trimmed chlorophyte BUSCO genes used for phylogenomics analyses.

**Supplemental File S10.** IQ-TREE phylogeny produced from chlorophyte BUSCO genes.

**Supplemental File S11.** ASTRAL-III phylogeny produced from chlorophyte BUSCO genes.

**Supplemental File S12.** *Chlamydomonas reinhardtii*–*C. incerta* synteny blocks.

**Supplemental File S13.** *Chlamydomonas reinhardtii*–*C. incerta* syntenic orthologs.

**Supplemental File S14.** *Chlamydomonas reinhardtii*–*C. schloesseri* synteny blocks.

**Supplemental File S15.** *Chlamydomonas reinhardtii*–*C. schloesseri* syntenic orthologs.

**Supplemental File S16.** *Chlamydomonas reinhardtii*–*E. debaryana* synteny blocks.

**Supplemental File S17.** *Chlamydomonas reinhardtii*–*E. debaryana* syntenic orthologs.

**Supplemental File S18.** Chlorophyte *L1* LINE proteins.

**Supplemental File S19.** Aligned and trimmed chlorophyte *L1* LINE proteins.

**Supplemental File S20.** IQ-TREE phylogeny of chlorophyte *L1* LINE proteins.

**Supplemental File S21.** OrthoFinder gene clustering of six core-*Reinhardtinia* species.

**Supplemental File S22.** InterProScan summary for genes of six core-*Reinhardtinia* species.

**Supplemental File S23.** InterPro domains associated with core-*Reinhardtinia* orthogroups.

**Supplemental File S24.** Coding potential metrics and low coding potential gene set. The 250 genes that failed all three tests are labelled “low_coding_potential_1” and the 471 genes that failed PhyloCSF and one other test are labelled “low_coding_potential_2”.

**Supplemental File S25.** InterProScan raw output for novel *C. reinhardtii* genes.

**Supplemental File S26.** phastCons CEs in *C. reinhardtii* v5 coordinates.

**Supplemental File S27.** UCEs in *C. reinhardtii* v5 coordinates.

## Supplementary Material

koab026_Supplementary_Data

## Notes

R.J.C. performed analyses and wrote the first draft of the manuscript, with the exception of the analyses and manuscript section on mating-type evolution, which were performed and written by A.R.H. R.J.C. and R.W.N. performed laboratory work. R.J.C., R.W.N., and P.D.K. conceived the study. All authors read and commented on the final draft version of the manuscript.

The author responsible for distribution of materials integral to the findings presented in this article in accordance with the policy described in the Instructions for Authors (https://academic.oup.com/plcell) is: Rory J. Craig (rory.craig@ed.ac.uk).
