# Supplementary material for: Comparative genomics of Chlamydomonas
Source: Plant Cell. 2021 Feb 2;33(4):1016–41. doi: 10.1093/plcell/koab026 (PMC8226300; doi:10.1093/plcell/koab026)
Supplement: koab026_Supplementary_Data [file koab026_supplementary_data.zip › tpc.00468.2020-s02.pdf]

Comparative genomics of *Chlamydomonas*

Rory J. Craig, Ahmed R. Hasan, Rob W. Ness & Peter D. Keightley

Corresponding author: Rory J. Craig rory.craig@ed.ac.uk

Review timeline:

**TPC2020-RA-00468**	Submission received:	Jun. 16, 2020
	1st Decision:	Aug. 13, 2020 *request revisions*
**TPC2020-RA-00468R1**	1st Revision received:	Oct. 26, 2020
	2nd Decision:	Dec. 11, 2020
	Final acceptance:	Jan. 21, 2021

REPORT: (The report shows the major requests for revision and author responses. Minor comments for revision and miscellaneous correspondence are not included. The original format may not be reflected in this compilation, but the reviewer comments and author responses are not edited, except to correct minor typographical or spelling errors that could be a source of ambiguity.)

TPC2020-RA-00468 1st Editorial decision – *request revisions* Aug. 13, 2020

We have received reviews of your manuscript entitled "Comparative genomics of Chlamydomonas." Thank you for submitting your best work to The Plant Cell. The editorial board agrees that the work you describe is substantive, falls within the scope of the journal, and may become acceptable for publication, pending revision and potential re-review. Members of the editorial board feel that the revised manuscript could fit nicely in our "Biology of Plant Genomes" focus issue - see https://aspb.org/publications/tpcfocus/ .

Papers to be included in the Biology of Plant Genomes focus issue (and TPC more generally) must go beyond purely descriptive analyses of genome content and structure. As you will see in the reviewer comments, the submitted manuscript nicely describes comparative analyses of *C. reinhardtii* with the *Edaphochlamys debaryana*, *C. incerta* and *C. schloesseri* genomes you have generated, but we would like to see more extensive consideration of how comparative analyses advance understanding of the biology of all four lineages. I suggest that you dive deeply into one or two of the topics mentioned by reviewers #1 and #2. Please read and respond to the comments of all three reviews and let us know if you have any questions about how you might address the reviewer concerns and/or adjust the frame of the manuscript to fit within the scope of our "Biology of Plant Genomes" focus issue.

TPC2020-RA-00468R1 1st Revision received Oct. 26, 2020

--- Reviewer comments:

Reviewer #1 (Comments for the Author):

Point 1. Section starting on line 615. Is there a Supplemental table of genes "lost" from *C. reinhardtii* in the recent annotation upgrade and those recovered in here? If not there should be.

RESPONSE: Thank you for highlighting this oversight, we have now included this data in Supplemental Table 12. We have also revised the number of such genes from 90 to 35, with the higher original count caused by an unfortunate coding error. The issue of "lost" genes was recently documented by Blaby and Blaby-

Haas (2017), who highlighted the *psbW* gene as a particularly egregious case. We were happy to see that *psbW* is included in our recovered set (gene ID: g7740.t1). It may also be interesting to note that there were functionally characterised genes in the "novel" set that were absent from both v4 and v5, such as *REX1-S* (g1146.t1). In the case of *REX1-S* it was likely consistently missed because it is dicistronic (Cenkci, et al. 2003), which is a nice example of the interesting things you can discover (or re-discover) with a comparative approach.

Point 2. Analysis of expanded gene families in *E. debaryana* starting on line 488. Table S9. Despite the authors' argument that these data cannot be understood without more information on the biology of *E. debaryana*, I would argue the opposite. Use the data to try and generate hypotheses. Among the ortho-groups, I noticed some repeated or overlapping annotations for the ones with domains (e.g. SRCR domains, transcription factors). I suspect that an enrichment analysis could reveal patterns that at least would provide a hint about what types of biological processes are associated with the OG expansions. Moreover, the authors could also make use of data from *C. reinhardtii* by mining information such as GO or domain term enrichment of its equivalent set of genes from the OGs which are expanded in *E. debaryana*.

RESPONSE: This is an important comment. We have attempted to add more discussion on both *the E. debaryana* expanded families and the *Chlamydomonas*-specific families (lines 410-459), although lacking a thorough grounding in protein function I am unsure how interesting these observations are. One mildly noteworthy result that came from a closer look is that most of the diversity in the NCL gene family, which is experiencing an ongoing expansion and diversification in *C. reinhardtii* (Boulouis, et al. 2015), is limited to *C. reinhardtii* itself and presumably has some specific (and currently unknown) relationship with the biology of *C. reinhardtii* relative to *C. incerta* and *C. schloesseri*.

In general, my limited understanding is that although many of these genes have functional domains, we don't actually know what most of them are doing in chlorophytes. From conversations with more functionally minded colleagues my impression is also that GO terms in Chlamydomonas are available for a minority of genes and in several cases may be misleading due to liftover from plant or animal functions. For example, the *SRCR* and C-type *lectin* genes that are enriched in *E. debaryana* are also a large gene family in *C. reinhardtii* (Wheeler, et al. 2008), although as far as I can tell there is only an implication that they may be involved in innate immunity as that is their major role in animals. None of the *C. reinhardtii* orthologs of the *E. debaryana* expanded families had specific gene names and verified functions. I would be happy to attempt a more formal statistical analysis, but my instinct is to be very cautious when we know essentially nothing about the biology of these species.

Point 3. Formally speaking, it cannot be said with certainty that a *MT* locus has been identified for any of the three newly sequenced species unless it can also be established that these species are heterothallic and not homothallic. It is expected that the *MID* gene would be retained even in homothallic species, so its presence cannot be used as an argument for a mating locus (nor can the absence of the *FUS* gene which may be dispensable as it appears to be in *V. carteri*). One analysis that might support heterothallism would be unusual codon usage in *MID* or other candidate mating related genes like *MTD* which would not undergo meiotic recombination or gene conversion if they were in a heterothallic *MT*.

RESPONSE: An excellent and very important point. We were mainly working from the uncited assumption of heterothallism for *C. incerta* based on the low codon adaptation index for *MID* found by Popescu, et al. (2006). *E. debaryana* has at least two heterothallic mating pairs in culture. We had no grounds for assuming *C. schloesseri* was heterothallic.

We performed an additional analysis to quantify codon usage genome-wide for both *C. incerta* and *C. schloesseri* (lines 494-513, Supplemental Figure 7). For both species, *MID* is within the lowest ~2% of genes for its index of elongation (I_{TE}) value, a metric similar to the codon adaptation index. *MID* also had the lowest I_{TE} of any gene within the putative sequences syntenous to *C. reinhardtii MT*. *MTD1* had a low I_{TE} value in *C. schloesseri*, although not a particularly low value in *C. incerta*. We therefore proceed with the assumption (that should be verified in the future) that both species do have *MT* loci, although *MTD1* may no longer be *MT*- limited in *C. incerta*.

Point 4. Section starting line 631. It would be nice to show a *C. reinhardtii* genome browser shot figure highlighting different kinds of ultraconserved elements.

RESPONSE: Would it be possible to provide more detail on what you would like to see and what is meant by different kinds of UCEs? Almost all of them entirely overlap coding sequence, but perhaps it may be informative to show a browser shot of a gene with a UCE and several CEs, showing the accompanying alignment to the other species?

Point 5. It would be a shame if these data did not end up being hosted in Phytozome/Phycocosm at JGI. Are the authors planning to discuss this with JGI personnel? If not will they be hosting their own public genome browsers?

RESPONSE: The genomes have been uploaded to Phycocosm and are currently available for download and associated genome browser analyses. There is currently no plan to implement them in Phytozome, although this would be very attractive if the opportunity presented itself.

Point 6. Line 460. Please clarify this sentence. The authors intent is to describe HR rates in vegetative/mitotic phase cultures. They need to specify the life cycle phase since HR occurs at normal rates during meiosis.

RESPONSE: This is a very interesting point, although with brevity in mind we have not currently extended this section. We would argue that even if HR is frequent in meiosis this would have a negligible impact on overall genome evolution, as from the very little *C. reinhardtii* ecology that is known we expect the species to undergo many clonal generations for every sexual event. This was recently estimated as one sexual cycle for every ~840 generations (Hasan and Ness 2020). It may be more important if HR is more frequent during the mitotic cell cycle phases where the sister chromatids are present, although I'm unaware if this has been shown in *C. reinhardtii*.

Point 7. Section on intron lengths starting line 665. The authors describe volvocine introns as atypically long, but their comparative data for "normal" intron length seems biased to me. Most of it comes from one taxonomic group, opisthokonts, and/or model organisms where genome size was a factor in selection of the species as a model.

RESPONSE: In this case we would cautiously argue that it is necessary to focus on species where genome size is a factor. The aim of the comparison is to attempt to compare species that have similarly compact genomes (as approximately as that can be achieved across such distantly related species). We want to compare compact genomes as the intron lengths are presumably under selection to be a particular length, as has been suggested in Drosophila (Halligan and Keightley 2006). If larger genomes are included, then intron length may be dominated by drift and possible repeat expansions. The atypical nature of introns in Chlamydomonas is that they have a tight distribution around a length that is much longer than usually seen in similarly compact genomes. This was highlighted in the original genome paper (Merchant, et al. 2007).

With respect to the taxonomic limitation, this is a valid criticism. The brown alga *Ectocarpus* was specifically included as an example of another species (that is not a plant of opisthokont) that has longer introns. Many of the other appropriate model systems (e.g. *Chondrus crispus* and

***Phaeodactylum tricornutum*) have so few introns that they cannot be included. We would be happy to include additional species, although we generally stand by the observation that the long introns of Chlamydomonas (and *Ectocarpus*) are interesting and unexplained genomic features.**

Point 8. The premise about intron length and conservation starting on line 668 seems convoluted or flawed. The enrichment of CEs within short introns may just be a reflection of some minimal fixed length of CEs within all introns, with any additional sequences beyond that minimum evolving with few constraints. This to me seems like a reasonable default idea to start with and requires no additional assumptions about a specific relationship between intron size and regulation.

RESPONSE: This is absolutely correct. Having looked into this further there is no significant difference between shorter and longer introns in terms of the total amount of CE sites. The abstract and discussion section has been changed to reflect this.

Point 9. Line 720. Many RP genes bind RNA. One additional (and perhaps primary) reason RP genes are enriched for UCEs involves autoregulation of RP gene mRNA to control translation.

RESPONSE: Thank you for bringing this interesting biological feature to our attention. We have included one sentence on this possibility (line 794-796).

Reviewer #2 (Comments for the Author):

Point 1. As for the specific points, I do not agree on one of the remarkable claims (mentioned even in the abstract) that *C. reinhardtii* experienced a recent degeneration event based on the genome assembly size (111.1 Mb) compared to the newly sequenced relative assemblies (*C. incerta*: 129.2 Mb; *C. schloesseri*: 130.2 Mb). *C. reinhardtii* genome was done by Sanger sequencing which was vulnerable to repetitive regions and tended to underestimate them. Because SMRT sequencing is an emerging technology, the authors should be very careful when they compare assemblies of different strategies.

RESPONSE: This is an important point and something we spent some time considering when writing the first draft. However, we think the claims of larger genome sizes are robust. The *C. reinhardtii* assembly was Sanger sequenced, however the assembly gaps consisting of Ns are included in the assembly size. To my understanding, the gap lengths were either estimated based on knowledge of the insert size (in the BAC, fosmid, etc.) or more roughly from physical recombination data for larger gaps between scaffolds. Although it is of course possible that the true genome size of *C. reinhardtii* is one or two megabases larger than the assembled size, we think it would be highly unexpected to find that it was ten or twenty megabases larger.

I have also been fortunate enough to be involved with the assembly of four long-read genomes for different *C. reinhardtii* strains this year, all of which come out at ~110 Mb, so it is not a feature of differences in sequencing technology. Having worked on these assemblies I can also say that *C. reinhardtii* version 5 is actually quite complete with respect to transposable elements (which Sanger would be expected to have less trouble with), with the majority of assembly gaps corresponding to microsatellite and satellite DNA. One possibility with long-read assemblies is that there may be some redundancy in the most repetitive and highly fragmented contigs, however even if we only consider contigs that are syntenous between *C. incerta/C. schloesseri* and *C. reinhardtii* then the latter

assemblies are still larger (lines 252-255). More genomes will certainly be needed to comment on genome size evolution in the lineage, but we believe it is correct to say that *C. reinhardtii* is likely to have the smallest genome of the eight sequenced core-*Reinhardtinia* species.

Reviewer #3 (Comments for the Author):

Point 1. Please insert the specific strains that you used in the introduction (Lines 146-7).

RESPONSE: This has been added.

Point 2. Are the datasets used for phylogenetic analysis going to be deposited in a public repository. Sometimes, journal supplementary materials are not available after some time.

RESPONSE: We currently have not done so as there are very many supplementary files and datasets that would be available through the journal if accepted (following their guidelines). All code to produce these datasets and re-run phylogenetic analyses are available on Github, and presumably if one wanted to add additional species then they would have to re-run everything in any case.

Reviewers #1 and 2 (related comments for the Author):

Point a:

Reviewer 1: I completely understand that a genome (or genomes) paper is not usually meant to dive deep into any one topic, but it would be nice if the authors could do a little bit more on at least one of their analyses to provide a proof-of-principle example illustrating the advances one can make with multiple related genomes. This could be from any number of angles. For example-- conservation of miRNA genes or conservation of long non-coding mRNA genes, identification of conserved promoter elements for genes whose regulatory elements in *C. reinhardtii* are known, or even a proposed reconstruction of the history of the mating locus region in *C. reinhardtii* based on data from *C. incerta* and *C. schloesseri*.

Reviewer 2: Generally, however, I hesitate to recommend this manuscript now to the Plant Cell publication. Although at this moment the manuscript reads very well in molecular and genomic evolution, I am not totally sure it fits the Plant Cell as the most prestigious journal in the plant physiology field. The authors claim that the genome-level evolution shown here provides insights into green algal functional conservation and boosts the researches in this field. I think they should demonstrate its potential by showing at least one novel functional (could be physiological, developmental, or ecological) investigation based on screening the assembled genomes. Without that, it is difficult for the general Plant Cell readers to realize the idea that this manuscript contributes to the wider plant physiologist community.

RESPONSE: Thank you for these suggestions and for giving us the opportunity to include an additional analysis. We spent some time considering what would be the best area to target. I was interested in looking further at the conservation of specific features such as known regulatory elements or RNA genes, however in these cases we are currently limited by phylogenetic power. The conserved elements are generally far longer (>100 bp) than small RNA genes and certainly individual regulatory motifs, and thus although one would expect an enrichment of CEs at such sites (and there does seem to be from a

preliminary check of small RNA genes from NCBI), it is not possible to go much further beyond that. We would require several additional close relatives of *C. reinhardtii* to first be discovered and then sequenced to achieve the appropriate phylogenetic power to detect shorter and more specific conserved elements from the whole-genome alignment. This has been achieved for Arabidopsis, Drosophila etc. and it is something I am very keen to pursue for *Chlamydomonas* in the future.

Instead, we decided to focus on further refining the gene annotations for *C. reinhardtii*, which obviously underlie almost all aspects of modern genetics research in the species. Alongside identifying conserved elements, improving gene annotation has been perhaps the foremost application of nucleotide-level comparative genomics. Whole-genome alignments have been used to great success in many species to distinguish between coding and noncoding sequences, with applications including the discovery of new genes or exons, scoring candidate long noncoding RNA genes for potential protein-coding capacity, and the identification of non-standard gene features such as polycistronic genes and stop codon readthrough (see the addition to the first paragraph of the introduction). Unlike conserved elements, this does not require as much phylogenetic signal in the alignment, as coding sequence has very specific evolutionary characteristics that can be assessed even amongst only a small number of species (such as faster evolution at putative synonymous sites vs nonsynonymous sites).

We already had the short section on the discovery of novel genes in *C. reinhardtii*, and we decided to extend this by performing a more thorough assessment of coding potential across all C. reinhardtii v5 gene models. When the v5 annotation of *C. reinhardtii* was produced, the only available comparisons were at the protein-level (to species such as *Volvox*). As a result, there are more than 2,000 genes in the annotation that have no homology support and are based entirely on ab initio gene prediction. Many of these genes do now have homology support from our new annotations, but there remain more than 1,000 genes that do not and are therefore of questionable protein-coding potential. It is possible that many of these genes are false positives based on spurious long ORFs, which are not unexpected in a species with such high GC content (and thus fewer stop codons in random sequence). We used a comparative analysis to identify more than 800 genes that have no homology support and showed no evidence for evolving under protein-coding constraint based on the whole-genome alignment. We augmented this analysis with recently produced genetic diversity data, showing that around ~600 of the above genes are extreme outliers with respect to the ratio of genetic diversity at zero-fold and four-fold degenerate sites (i.e. they are also not evolving under protein-coding constraint in a population context). We finally included codon adaptation data to show that many of these genes also possess codon usage patterns that deviate from the overall codon usage of *C. reinhardtii* genes. As a proof of principle, we took the intersection of all three analyses as a high confidence false positive set (250 genes) and demonstrated that these genes are almost certainly not protein coding using patterns of genetic diversity at high-impact sites (start codons, stop codons and splice junctions) and start codon sequence context (i.e. the genes lack recognisable Kozak sequences).

We think that this analysis shows the strength of comparative genomics and we hope that it serves as a good example of how our new resources can be used in a more directly functional sense. Indeed, I am currently involved in updating the *C. reinhardtii* genome assembly and annotation and I am using these resources for this very purpose, adding new protein coding genes and removing false positive models. We expect that a more accurate annotation will aid in several areas. For example, long noncoding RNA genes are greatly understudied in *C. reinhardtii*, and it is possible that many of the false positive models are in fact long noncoding RNA genes that happen to contain long ORFs by chance.

References

Blaby IK, Blaby-Haas CE. 2017. Genomics and functional genomics in Chlamydomonas reinhardtii. In: Hippler M, editor. Chlamydomonas: Molecular genetics and physiology: Springer.
Boulouis A, Drapier D, Razafimanantsoa H, Wostrikoff K, Tourasse NJ, Pascal K, GirardBascou J, Vallon O, Wollman FA, Choquet Y. 2015. Spontaneous dominant mutations in Chlamydomonas highlight ongoing evolution by gene diversification. Plant Cell 27:984-1001.
Cenkci B, Petersen JL, Small GD. 2003. REX1, a novel gene required for DNA repair. J Biol Chem 278:22574-22577.
Halligan DL, Keightley PD. 2006. Ubiquitous selective constraints in the Drosophila genome revealed by a genome-wide interspecies comparison. Genome Research 16:875-884. Hasan AR, Ness RW. 2020. Recombination rate variation and infrequent sex influence genetic diversity in Chlamydomonas reinhardtii. Genome Biol Evol.
Merchant SS, Prochnik SE, Vallon O, Harris EH, Karpowicz SJ, Witman GB, Terry A, Salamov A, Fritz-Laylin LK, Marechal-Drouard L, et al. 2007. The Chlamydomonas genome reveals the evolution of key animal and plant functions. Science 318:245-250.
Popescu CE, Borza T, Bielawski JP, Lee RW. 2006. Evolutionary rates and expression level in Chlamydomonas. Genetics 172:1567-1576.
Wheeler GL, Miranda-Saavedra D, Barton GJ. 2008. Genome analysis of the unicellular green alga Chlamydomonas reinhardtii indicates an ancient evolutionary origin for key pattern recognition and cell-signaling protein families. Genetics 179:193-197.

TPC2020-RA-00468R1 2nd Editorial decision – *acceptance pending* **Dec. 11, 2020**

We are pleased to inform you that your paper entitled "Comparative genomics of Chlamydomonas" has been accepted for publication in The Plant Cell, pending a final minor editorial review by journal staff. Thanks for your excellent care with the revisions! At this stage, your manuscript will be evaluated by a Science Editor with respect to its presentation of scientific content, compliance with journal policies, and presentation for a broad readership.

Final acceptance from Science Editor **Jan. 21, 2021**
