# Supplementary material for: Comparative genomics of Chlamydomonas
Source: Plant Cell. 2021 Feb 2;33(4):1016–41. doi: 10.1093/plcell/koab026 (PMC8226300; doi:10.1093/plcell/koab026)
Supplement: koab026_Supplementary_Data [file koab026_supplementary_data.zip › tpc.00468.2020-s04.pdf]

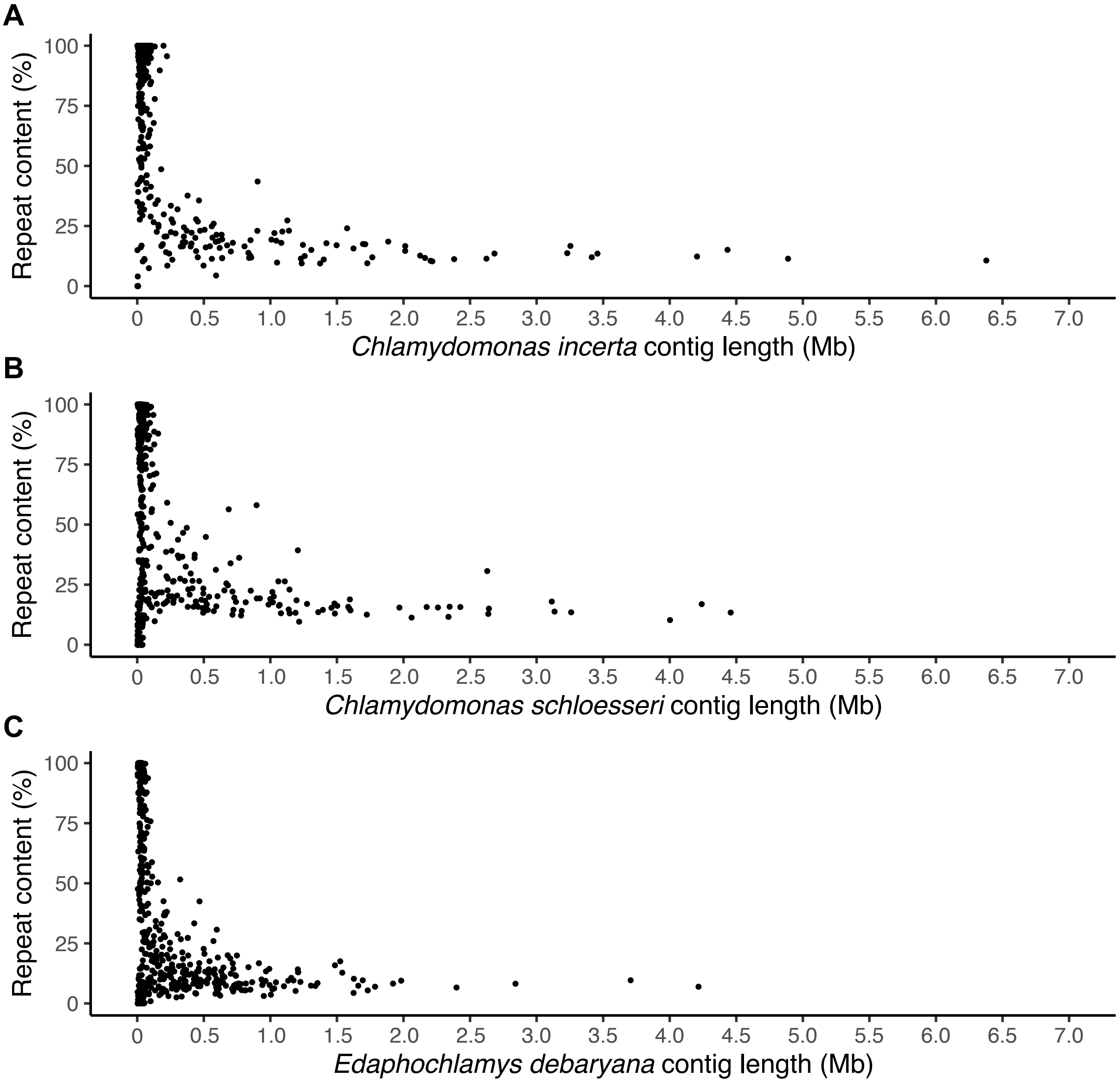

Supplemental Figure 1. Total repeat content per contig (transposable elements, satellites and simple/low-complexity repeats) plotted by contig length for *Chlamydomonas incerta* **(A)**, *Chlamydomonas schloesseri* **(B)** and *Edaphochlamys debaryana* **(C)**.
(supports Table 1).

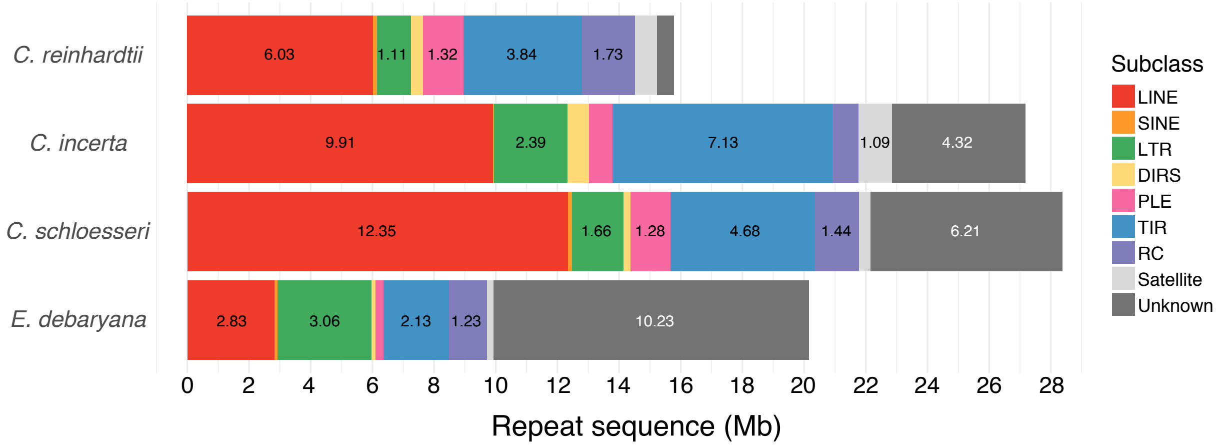

Supplemental Figure 2. Repeat content per species by repeat subclass.
Numbers within bars represent total sequence per subclass, in megabases (Mb). LINE = long interspersed nuclear element, SINE = short interspersed nuclear element, LTR = long terminal repeat, DIRS = tyrosine recombinase encoding retrotransposons, PLE = Penelope-like elements, TIR = terminal inverted repeat (i.e. DNA transposons), RC = rolling-circle elements. Note that the cumulative repeat content totals are marginally higher than those in Table 1 due to redundancy in repeat classification. (supports Table 1).

A

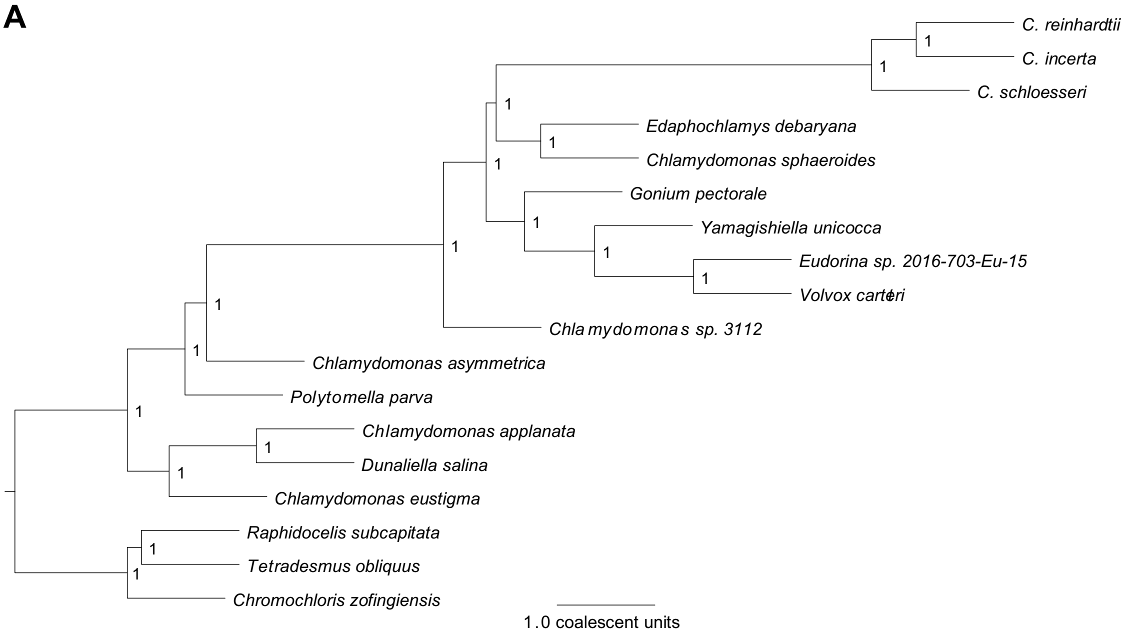

B

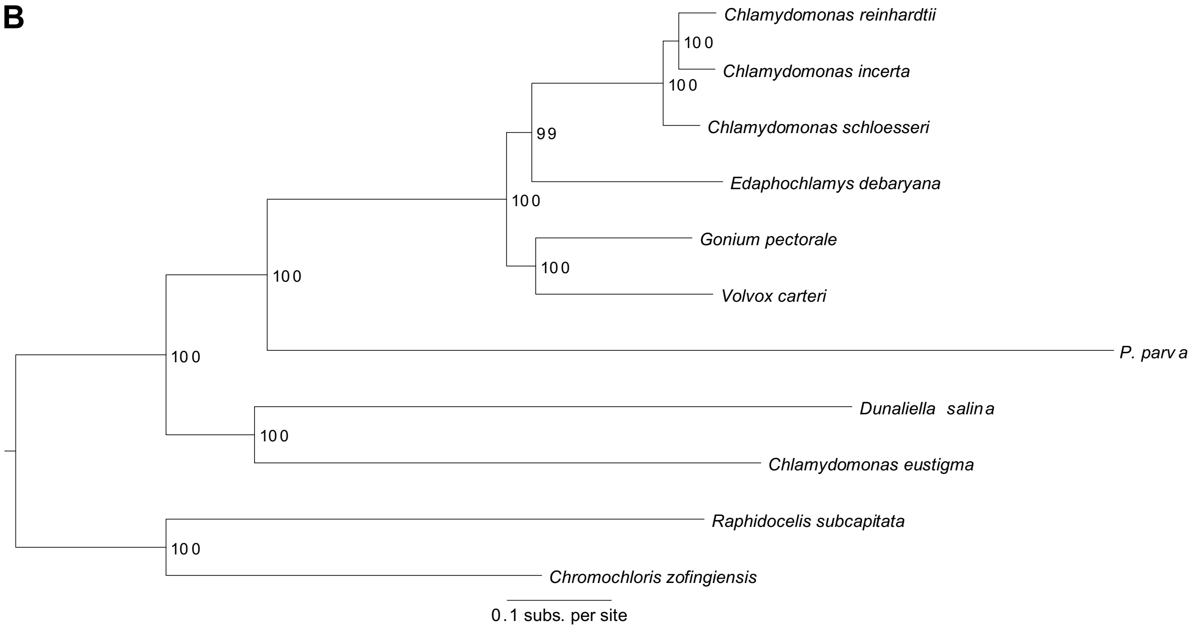

Supplemental Figure 3. Phylogenomic analyses.
(A) ASTRAL-III species tree (15 Volvocales species and three outgroups) summarizing 1,624 gene trees produced from individual protein alignments of chlorophyte BUSCO genes.
(B) ML phylogeny of nine Volvocales species and two outgroups inferred using LG+F+R5 model and a concatenated protein alignment of 1,681 putative single-copy orthologs identified by OrthoFinder. (supports Figure 2).

C

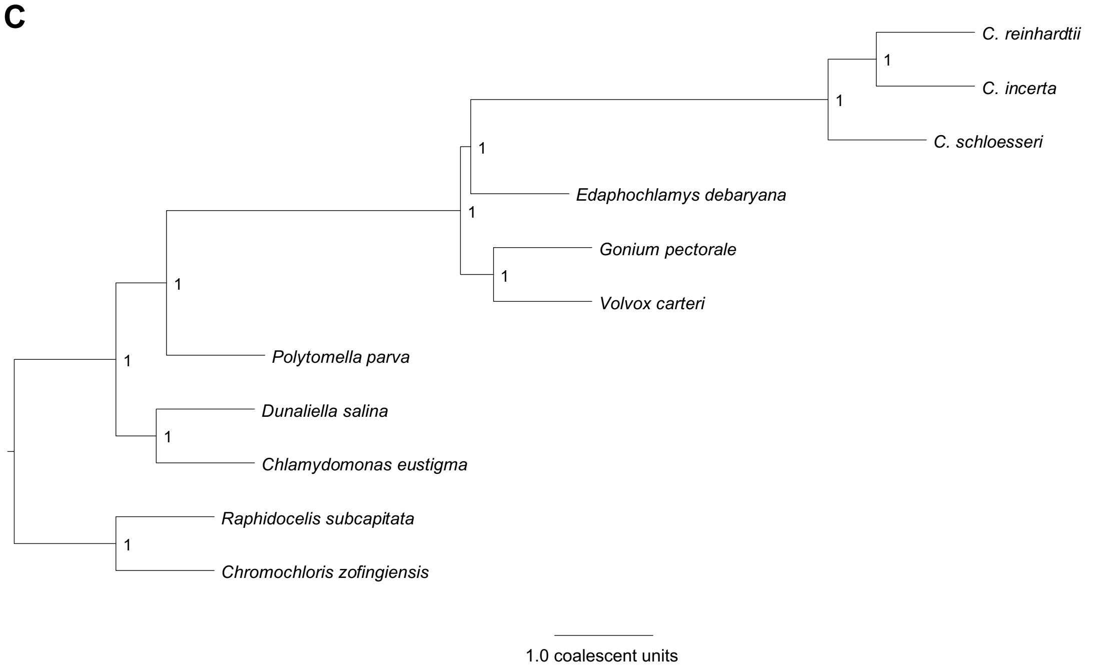

Supplemental Figure 3 (continued). Phylogenomic analyses.
(C) ASTRAL-III species tree summarizing 1,681 gene trees produced from individual protein alignments of the OrthoFinder single-copy genes.
Note that support values for (**A**) and (**C**) represent local posterior probabilities, while (**B**) represents ultrafast bootstrap values.
(supports Figure 2).

A

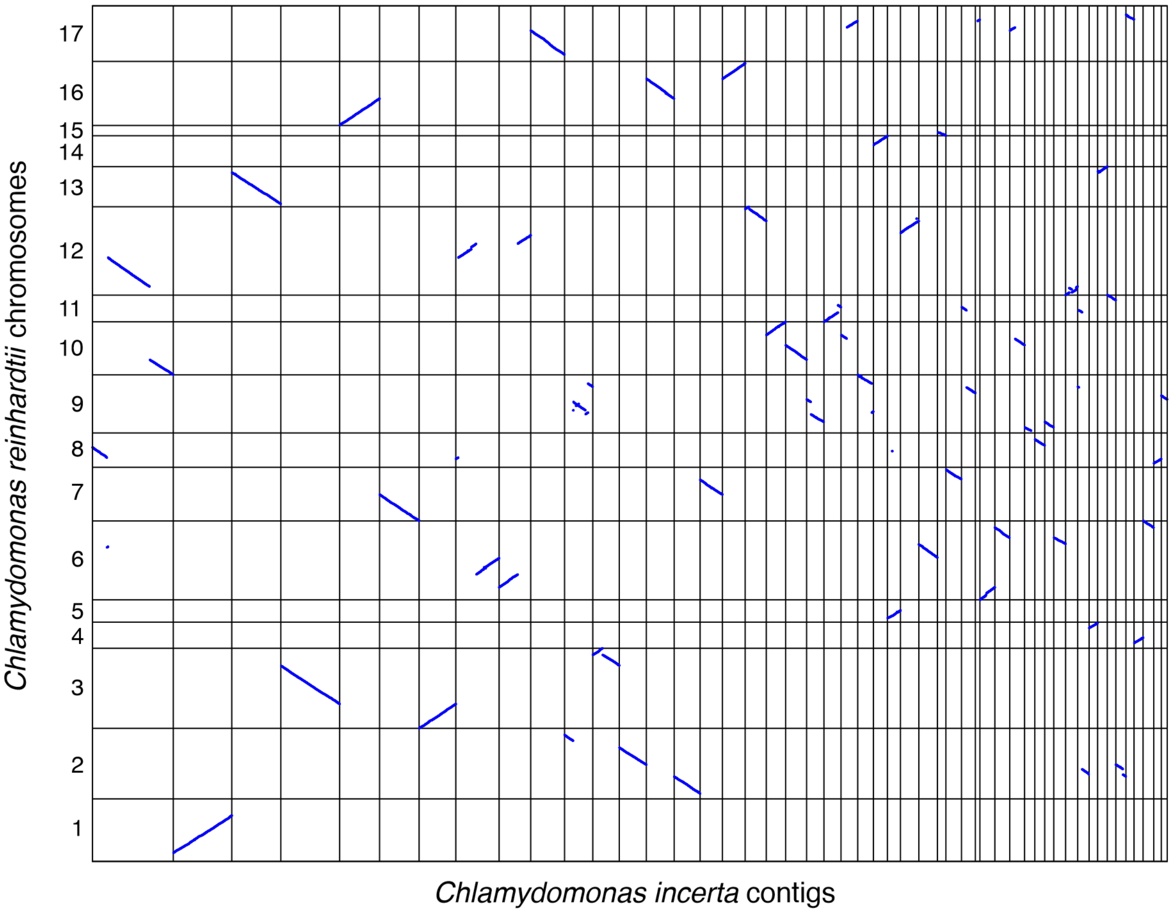

Supplemental Figure 4. Dotplots representing syntenic genomic segments identified between *C. reinhardtii* and 50 largest contigs of *Chlamydomonas incerta* **(A).**
(supports Figure 3).

B

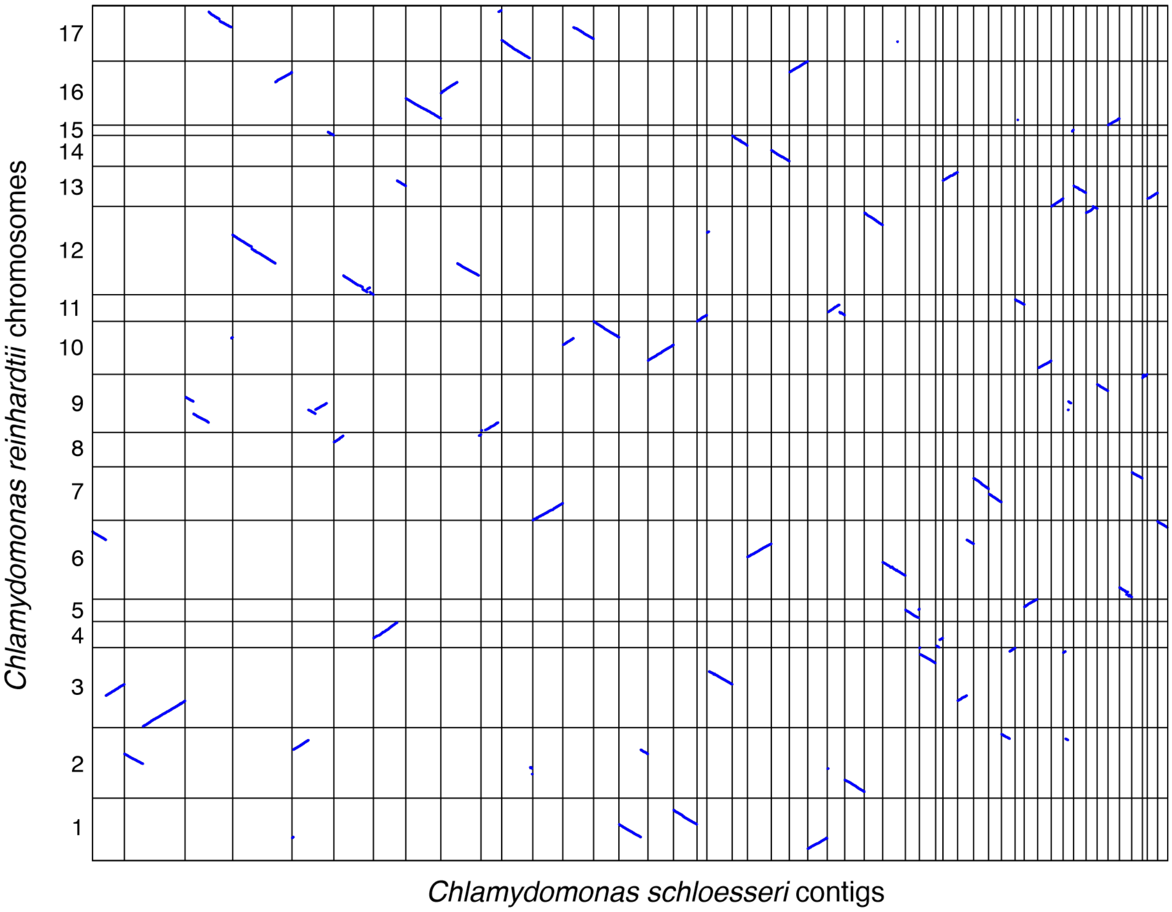

Supplemental Figure 4 (continued). Dotplots representing syntenic genomic segments identified between *C. reinhardtii* and 50 largest contigs of *Chlamydomonas schloesseri* **(B).**
(supports Figure 3).

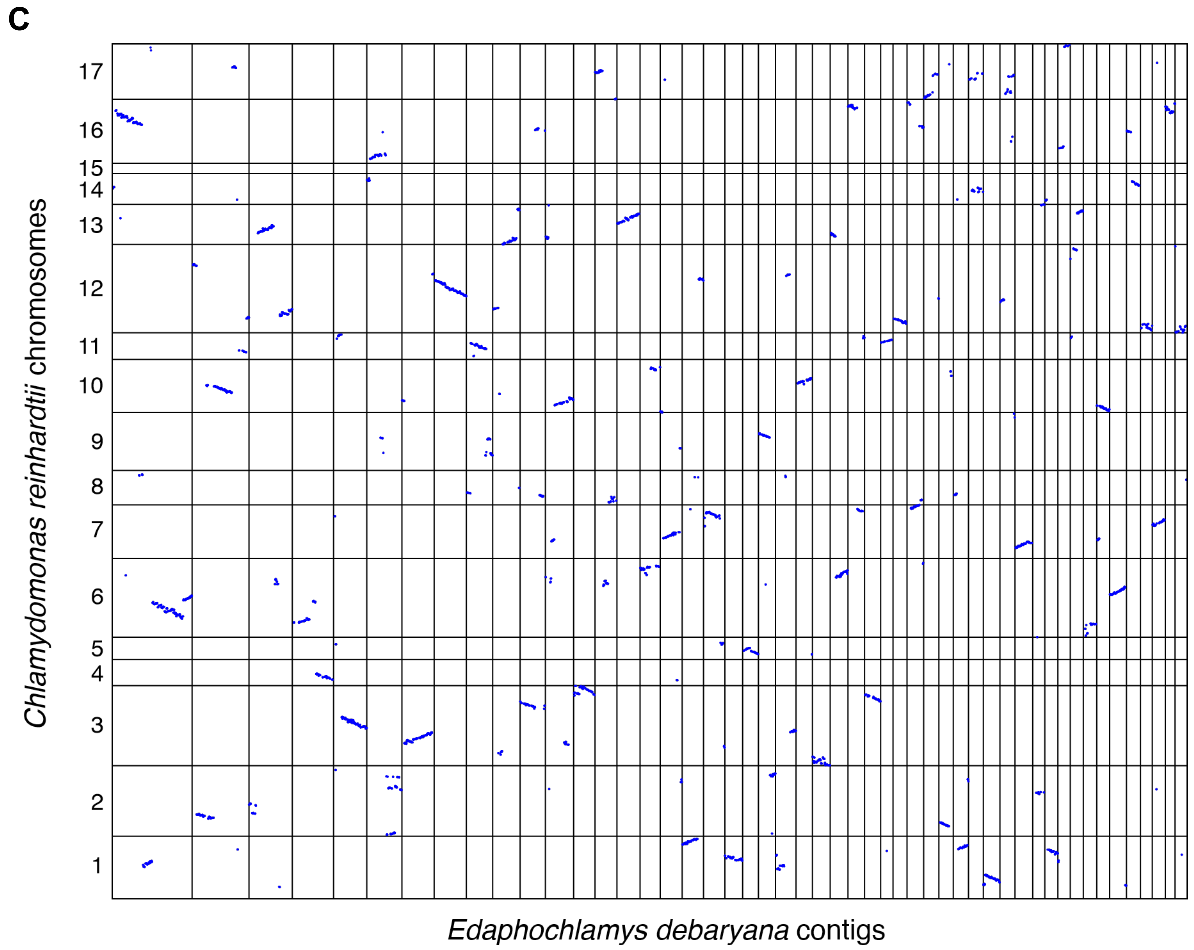

Supplemental Figure 4 (continued). Dotplots representing syntenic genomic segments identified between *C. reinhardtii* and 50 largest contigs of *Edaphochlamys debaryana* **(C)**.
(supports Figure 3).

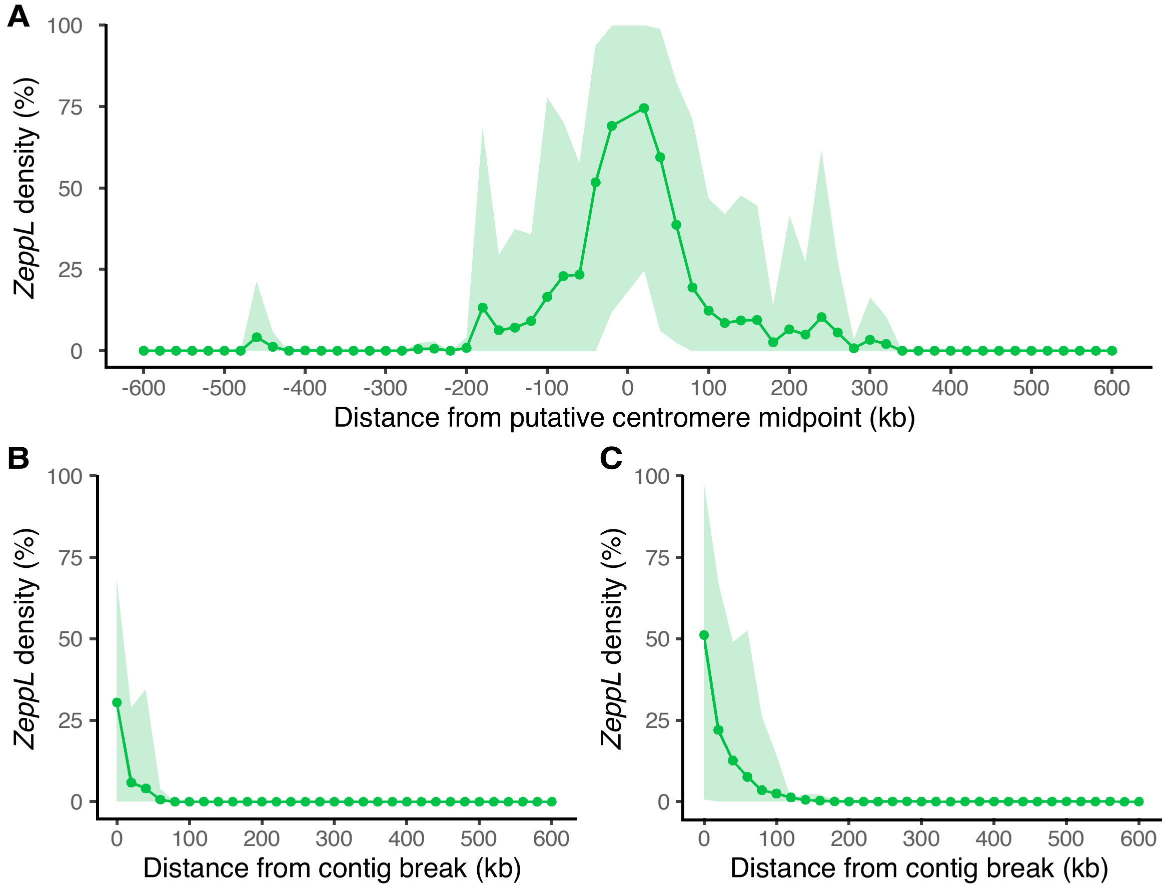

Supplemental Figure 5. Mean densities of *Zepp*-like *L1* LINE elements per 20 kb windows averaged over relevant chromosomes/contigs.
Shaded areas represent 95% quantiles.
(A) Density of *L1-1_CR* / *ZeppL-1_cRei* elements relative to midpoint of 15 putative *C. reinhardtii* centromeres.
(B) Density of *ZeppL-1_cInc* elements relative to *C. incerta* contig ends syntenic to *C. reinhardtii* putative centromeres.
(C) Density of *ZeppL-1_cSch* and *ZeppL-2_cSch* elements relative to *C. schloesseri* contig ends syntenic to *C. reinhardtii* putative centromeres.
(supports Figure 4).

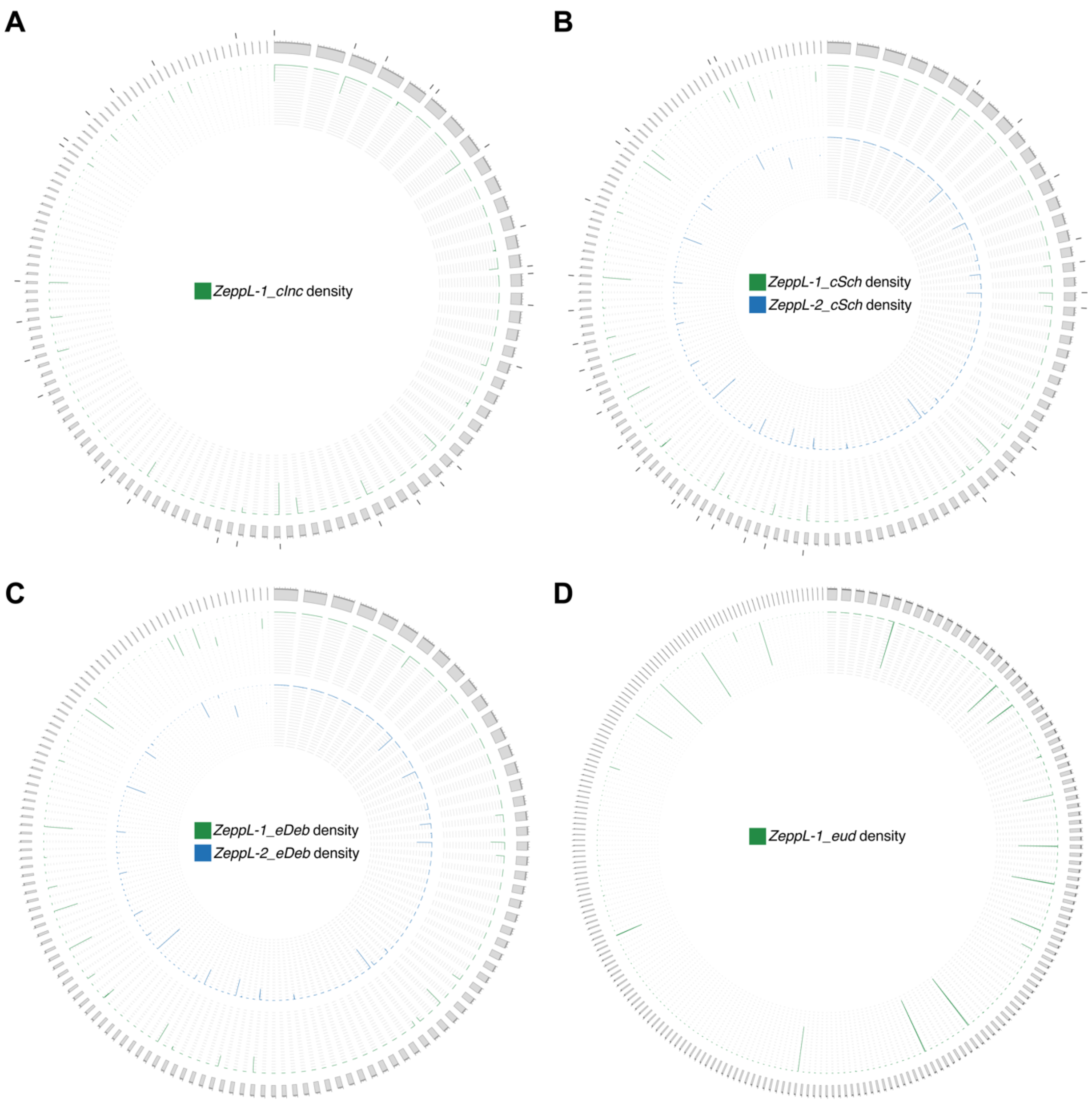

Supplemental Figure 6. Genome-wide density of *Zepp*-like elements.
Contigs are represented by gray bands and ordered by size. Dark gray ticks above/below contigs represent contig ends inferred as syntenic with *C. reinhardtii* centromeres. Axis ranges from 0-100% and densities calculated for 50 kb windows.
(A) *Chlamydomonas incerta*.
(B) *Chlamydomonas schloesseri*.
(C) *Edaphochlamys debaryana*.
(D) *Eudorina sp. 2016-703-Eu-15*.
(supports Figure 4).

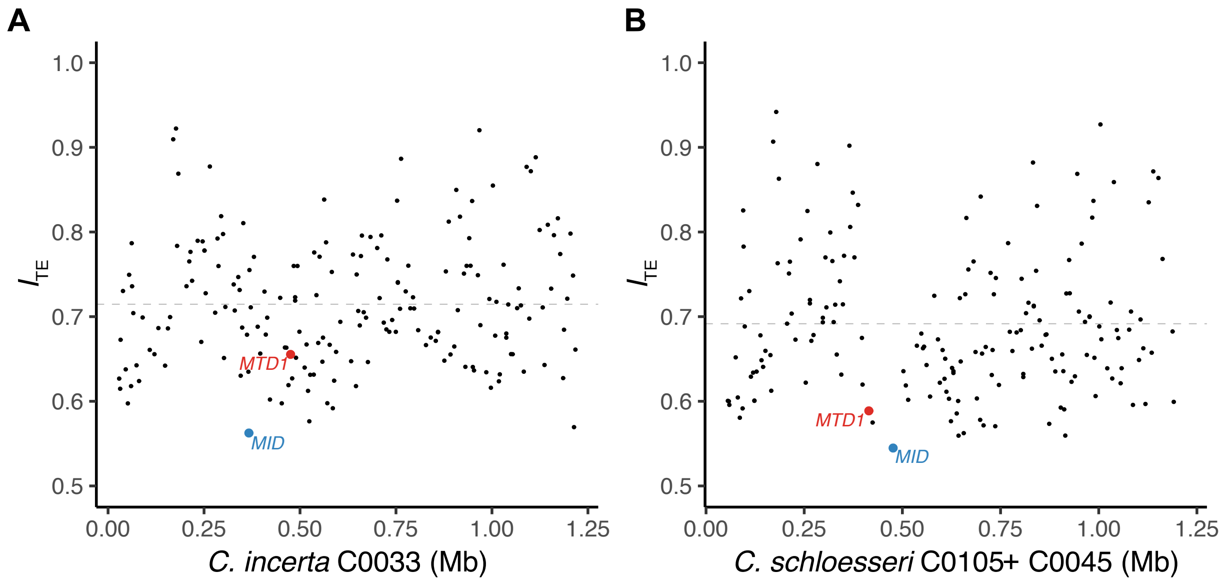

Supplemental Figure 7. Codon adaptation of *minus* mating type genes.
I_{TE} values are plotted for each gene across the putative minus mating type loci of *C. incerta* **(A)** and *C. schloesseri* **(B)**.
Each point represents a gene, with *MID* and *MTD1* orthologs highlighted. Dashed gray lines represent genome-wide means. Note that for *C. schloesseri* the region syntenous to the *C. reinhardtii* mating type is entirely on contig C0045, C0105 was appended to C0045 to show the genes syntenous with the most telomere-proximal region of *C. reinhardtii* chromosome 6.
(supports Figure 6).

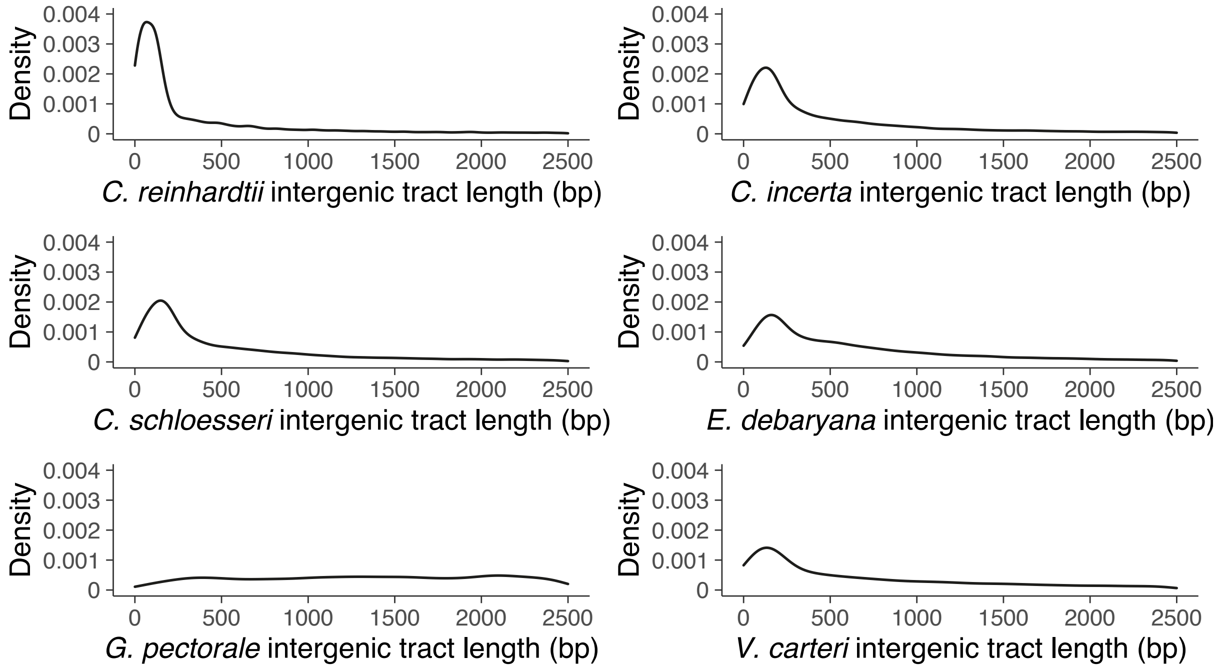

Supplemental Figure 8. Distribution of intergenic tract lengths across six core-*Reinhardtinia* species. The *G. pectorale* distribution likely differs due to the lack of UTR annotation for this species. (supports Figure 7).

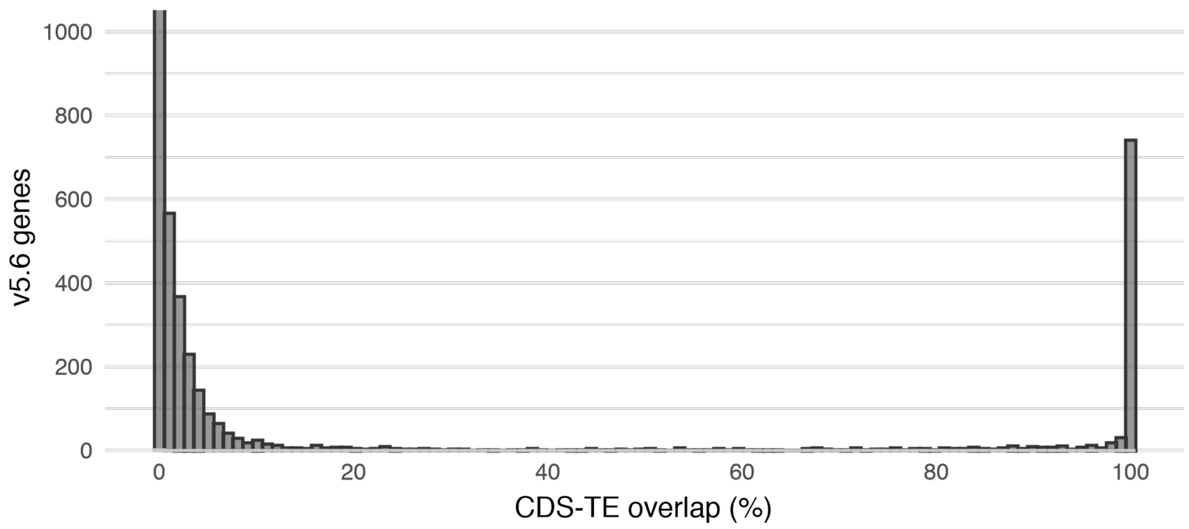

Supplemental Figure 9. Overlap between coding sequence of *C. reinhardtii* v5.6 genes and manually-curated *C. reinhardtii* transposable elements.
Note that the y-axis is cut at 1,000 genes and the majority of genes are therefore not shown (with 0% overlap).
(supports Figure 8).

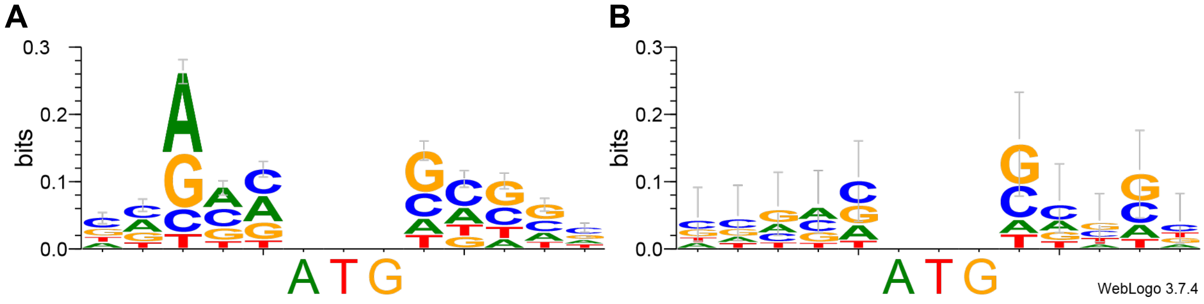

Supplemental Figure 10. Kozak consensus sequence logos.
(A) a randomly selected half of the control gene set. **(B)** the 250 low-coding potential genes that failed all three coding potential analyses (supports Figure 8).

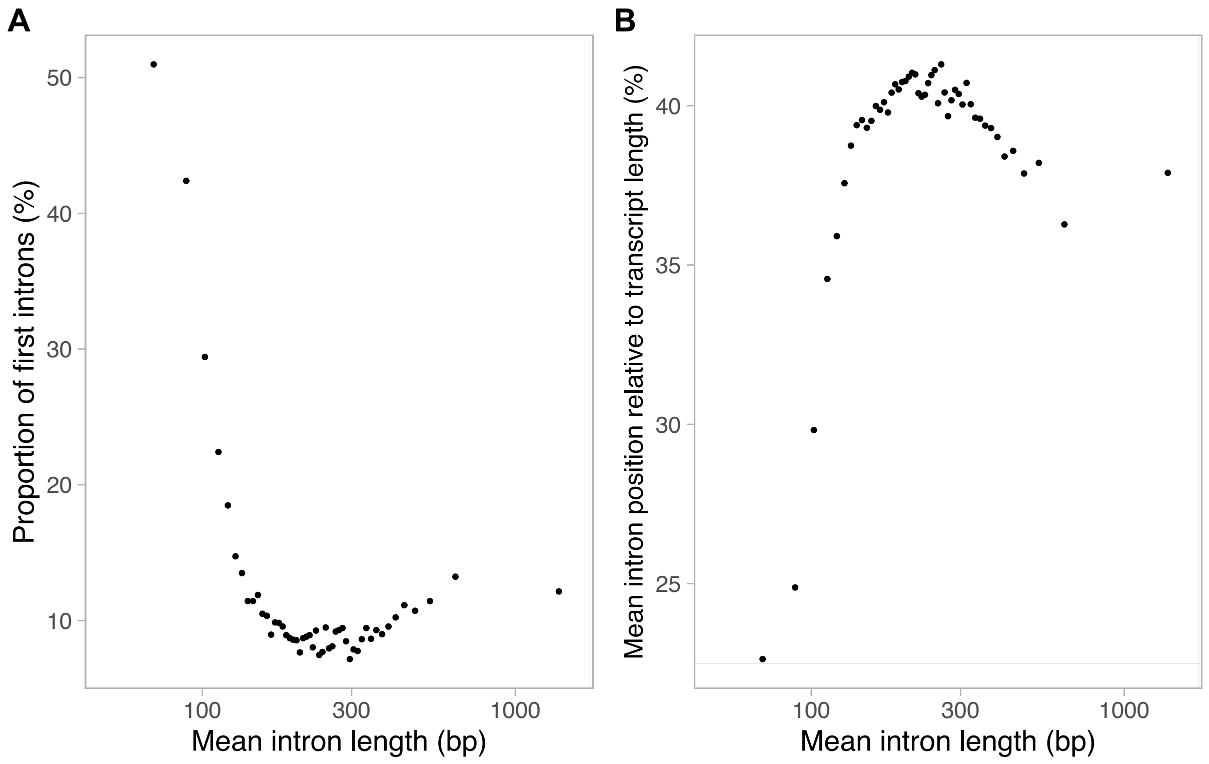

Supplemental Figure 11. Relationship between intron lengths and intron locations within genes.
(A) Relationship between the proportion of introns that are the first intron of a gene and the mean intron length per bin (see main text).
(B) The relationship between the mean intron position relative to transcript length (e.g. an intron at position 500 of a 2000 bp transcript equals 25%) and mean intron length per bin.
(supports Figure 9).
