# Supplementary material for: Comparative genomics of Chlamydomonas
Source: Plant Cell. 2021 Feb 2;33(4):1016–41. doi: 10.1093/plcell/koab026 (PMC8226300; doi:10.1093/plcell/koab026)
Supplement: koab026_Supplementary_Data [file koab026_supplementary_data.zip › tpc.00468.2020-s05.pdf]

Supplemental File 1. High molecular weight DNA extraction protocol

This protocol is a modified version of the *Chlamydomonas* high molecular weight DNA extraction protocol made available by the Joint Genome Institute:
https://www.pacb.com/wp-content/uploads/2015/09/DNA-extraction-chlamy-CTAB-JGI.pdf

The protocol is based on a CTAB and phenol:chloroform extraction, which is performed in two iterations to ensure maximum removal of RNA. The protocol should yield ~10 µg of high molecular weight DNA.

1. Inoculate a 6-well plate with *Chlamydomonas* from a slant culture in 5 mL Bold's basal medium per well and incubate on a plate shaker for 4 d. Up to nine wells can be used per sample (i.e. two plates and 45 mL total), but if higher yield is needed, perform multiple extractions per sample.
2. Preheat aliquot of CTAB buffer (see below) to 65ºC.
3. Transfer cultures to a 50 mL Falcon tube, centrifuge at 1,500*g* at room temperature to pellet cells and remove growth medium. Resuspend cells in 1 mL of lysis buffer (see below) by gentle pipette mixing and hand mixing in a swirling circular motion.
4. Add 1 mL of hot CTAB buffer, mix by gentle hand mixing and incubate at 65ºC for 30 min.
5. Decant the mixture into 15 mL phase-lock gel tube (e.g. QIAGEN MaXtract) and add 2 mL (~1 volume) room temperature phenol:chloroform:isoamyl alcohol (25:24:1). Gently mix by inverting ~40 times per min for 10 min.
6. Centrifuge for 8 min at 1,500*g* (or until phases are clearly separated) at room temperature. Transfer aqueous phase to a new 15 mL phase-lock gel tube.
7. Add 5 µL RNAse A. Gently mix and incubate at 37ºC for 30 min.
8. Repeat steps 5 and 6 (i.e. phenol:chloroform:isoamyl alcohol extraction).
9. Add 1 volume chloroform:isoamyl alcohol (49:1) (~2 mL), gently mix by inverting ~40 times per min for 10 min.
10. Centrifuge as in step 6 and transfer aqueous phase to a new 15 mL Falcon tube.
11. Add 2 volumes of ice-cold 100% ethanol, slowly mix by inverting 10 times end over end and incubate on ice for at least 1 h.
12. Carefully transfer the above mixture across four 1.5 mL DNA lo-bind tubes and centrifuge at 13,000*g* for 15 min at 4ºC. The DNA should be visible by eye, and in most cases the majority of the DNA will be in a single 1.5 mL tube as it would have formed a large clump in the previous 15 mL tube.

13. Discard supernatant and wash with 1 mL freshly prepared 70% ethanol (using DNAse-free water), invert gently and centrifuge at 13,000*g* for 1 min at 4ºC. Discard supernatant and repeat.

14. After removing most of the supernatant, briefly spin down washed pellets and remove any remaining ethanol by pipetting. Dry in flow hood for ~5 min (ensure all ethanol has evaporated but be careful not to over-dry pellet).

15. Resuspend pellets overnight in 45 µLTE buffer at 4ºC.

16. After checking that the DNA has completely dissolved, combine the contents of the four tubes by gently pipetting 45 µL from three of the tubes into the fourth tube (ideally pipetting into the tube containing the majority of the DNA to minimize shearing). Add 20 µL of 10x NEB3 buffer.

17. Add 8 µL of RNAse I_f, mix by gentle flicking and inversion, spin-down, and incubate for 30 min at 37ºC.

18. Add 2 µL of RiboShredder, incubate for a further 30 min at 37ºC.

19. Add 340 µL of DNAse-free water to bring total volume to 550 µL.

20. Add 550 µL phenol:chloroform:isoamyl alcohol (25:24:1), mix briefly and transfer to a 2 mL phaselock gel tube. As before, mix gently for 10 min and centrifuge for 8 min at room temperature at 14,000*g*.

21. Transfer to a second 2 mL phase-lock gel tube, add 1 volume chloroform:isoamyl alcohol (49:1), mix and spin-down as before.

22. Using a P1000 pipette, pipette 450 µL of the aqueous phase to a new 1.5 mL DNA lo-bind tube.

Add 50 µL of 3 M sodium acetate (pH 5.2) and mix gently by inverting.

23. Add 2 volumes of ice-cold ethanol (1 mL), precipitate and wash as in steps 12-14. Resuspend DNA pellet in 183 µL TE buffer overnight.

24. QC sample – Qubit 2 µL, Nanodrop 1µL. Pulse-field gel electrophoresis can be performed to check DNA fragment length distribution.

<u>Lysis Buffer (10 mL)</u>

50 mM Tris-HCl (pH 8.0) – 0.5 mL of 1 M stock
200 mM NaCl – 0.4 mL of 5 M stock
20 mM EDTA – 0.4 mL of 0.5 M stock
Nuclease-free water – 6.2 mL
2% SDS – 2 mL of 20% stock
Proteinase K (20 mg/mL) – 0.5 mL

<u>CTAB buffer (10 mL)</u>

50 mM Tris-HCl (pH 8.0) – 1 mL of 1 M stock
1.4 M NaCl – 2.8 mL of 5 M stock
20 mM EDTA – 0.4 mL of 0.5 M stock
2% CTAB – 0.2 g
1% PVP 40,000 – 0.1 g
Nuclease-free water to 10 mL
